# The SP1-SuperEnhancer-SPHK1 Axis Mediates Niraparib Resistance in TNBC

**DOI:** 10.3390/ph18111622

**Published:** 2025-10-27

**Authors:** Yu-Xia Yuan, Rui-Jia Chen, Gui-Hui Tu, Chao-Qi Li, Long-Long Xu, Yi-Ling Lu, Li-Xian Wu

**Affiliations:** 1 Department of Pharmacology, School of Pharmacy, Fujian Medical University, Fuzhou 350000, China; yyx19960829@163.com (Y.-X.Y.); ruijiachen503@163.com (R.-J.C.); tghinfuzhou@163.com (G.-H.T.); 18259630532@163.com (C.-Q.L.); 15120758581@163.com (L.-L.X.); 15859625230@163.com (Y.-L.L.); 2 Fujian Key Laboratory of Natural Medicine Pharmacology, Fujian Medical University, Fuzhou 350000, China

**Keywords:** niraparib, sphingolipid metabolism, SPHK1, super-enhancers, SP1

## Abstract

**Background**: PARP inhibitors exhibit significant lethality in tumors with BRCA1/2 mutations or homologous recombination defects; however, their clinical application is limited by the rarity of BRCA1/2 mutations, complex resistance mechanisms, and limited efficacy of monotherapy. **Objective**: This study aimed to investigate how Niraparib induces dysregulation of sphingolipid metabolism—particularly upregulation of the key enzyme SPHK1—in triple-negative breast cancer (TNBC) cells, and to elucidate a novel super-enhancer (SE)-mediated mechanism of Niraparib resistance. We also applied AI-based virtual screening to identify compounds targeting key nodes and develop strategies for sensitizing TNBC to Niraparib. **Results**: Niraparib induced sphingolipid metabolic imbalance and significantly upregulated SPHK1 in TNBC. Multi-omics analyses revealed that SPHK1 is regulated by a super-enhancer. Mechanistically, Niraparib inhibited PARylation of the transcription factor SP1, enhancing its occupancy at the SE region and reactivating its transcriptional activity, thereby promoting SPHK1 expression. This process activated pro-survival signaling pathways and conferred niraparib resistance. AI-facilitated virtual screening identified the natural compound Echinatin as a potent SP1 inhibitor, which stably binds to SP1 and exhibits marked synergistic effects with Niraparib. While targeting downstream SPHK1 also provided therapeutic benefit by enhancing the anti-tumor efficacy of Niraparib, Echinatin demonstrates a superior advantage due to its more favorable toxicity profile. **Conclusions**: Niraparib induces resistance through the SP1-SE-SPHK1 axis, whereas Echinatin effectively reverses this mechanism by inhibiting the upstream regulator SP1, significantly potentiating the efficacy of Niraparib. This study reveals a novel molecular mechanism underlying Niraparib resistance and proposes a promising combination therapy strategy.

## 1. Introduction

Triple-negative breast cancer (TNBC) is currently treated primarily with chemotherapy, and neoadjuvant chemotherapy combined with platinum-based agents can improve the pathological complete response rate (pCR), but the response rate remains limited and recurrence is common [1]. In recent years, immune checkpoint inhibitors (such as pembrolizumab) combined with chemotherapy have significantly improved survival in patients with PD-L1-positive metastatic TNBC [2], while targeted therapies (such as the PARP inhibitor Talazoparib) and antibody-drug conjugates (e.g., Sacituzumab govitecan) have provided new treatment options for specific subgroups, such as those with BRCA mutations or advanced TNBC [3,4].

PARP inhibitors significantly prolong progression-free survival (PFS) by selectively targeting TNBC cells with homologous recombination deficiency (HRD), such as BRCA mutations, while also offering the convenience of oral administration [5]. Four PARP inhibitors—Olaparib, Rucaparib, Niraparib, and Talazoparib—have been approved, marking breakthrough advancements in breast cancer treatment [6]. The four PARP inhibitors share a common “benzamide + aromatic fused-ring” scaffold. Differences in the ring systems, side-chain rigidity, and hydrogen-bond patterns fine-tune their potency, selectivity, and pharmacokinetic properties, thereby determining their clinical doses, indications, and adverse-effect profiles. Their advantages include high specificity for tumors with DNA repair defects and synergistic potential with chemotherapy or immunotherapy. However, significant challenges persist: widespread drug resistance due to restorative mutations or alternative DNA repair mechanisms (primary or acquired resistance); limited biomarkers, as only 15–20% of TNBC cases harbor BRCA mutations, and current diagnostics fail to fully identify other HRD subgroups [7]; and restricted efficacy of monotherapy, necessitating combination regimens that may increase hematologic toxicity [8,9].

In the process of tumorigenesis and progression, tumor cells primarily induce lipid metabolic reprogramming by influencing the uptake, synthesis, and breakdown of three major lipid molecules: cholesterol, fatty acids, and phospholipids. Breast cancer cells are surrounded by a large number of adipocytes, and lipid metabolism plays a crucial role in the growth of breast cancer cells [10]. Sphingolipid metabolism regulates cellular proliferation, apoptosis, and migration through signaling molecules such as ceramide and sphingosine-1-phosphate (S1P) [11]. In TNBC, dysregulated sphingolipid metabolism is characterized by reduced levels of pro-apoptotic ceramide and elevated pro-survival S1P, promoting tumor growth, invasion, and chemotherapy resistance [12]. Studies indicate that inhibiting sphingosine kinase 1 (SPHK1) or enhancing ceramide generation can improve TNBC sensitivity to chemotherapy [13], while aberrant expression of sphingolipid-related genes (e.g., ASAH1) correlates with poor prognosis in TNBC patients [14]. Targeting the sphingolipid metabolism pathway may emerge as a new strategy for treating TNBC. However, different lipids have varying effects on cells, and the roles of different molecules in sphingolipid metabolism are particularly complex, even opposing each other. Therefore, a “one-size-fits-all” approach to reducing lipid levels has limited anti-tumor effects. It is necessary to elucidate the specific mechanisms of sphingolipid alterations in order to achieve specific regulation of lipid metabolism and eliminate tumors.

Sphingosine kinase 1 (SPHK1) is a pivotal lipid kinase that catalyzes the phosphorylation of sphingosine (SPH) to sphingosine-1-phosphate (S1P). SPHK1 plays significant roles in diverse physiological processes, including cell proliferation and apoptosis, vasoconstriction and remodeling, inflammation, and metabolism [15]. Overexpression of SPHK1 is closely associated with tumorigenesis, progression, metastasis, and therapeutic resistance [16]. Molecularly, tumor development and progression are intricately linked to super-enhancers (SEs). SEs drive sustained transcription of pro-oncogenic genes and consist of clusters of highly active enhancers exhibiting greater transcriptional activity and higher cell-type specificity compared to typical enhancers [17]. SE regions are characterized by distinct epigenetic marks: H3K4me1, H3K27me3, and H3K27ac, representing quiescent, poised, and activated SE states, respectively, with H3K27ac serving as the most reliable marker for SE identification [17]. Previous studies have implicated SEs in metabolic reprogramming within tumors [18]. SEs, along with their transcription factors (TFs) and co-activators, form dense transcriptional regulatory complexes that potently enhance promoter and enhancer functions [19,20]. SPHK1 has been identified as an SE-associated oncogene in liver cancer, highlighting the impact of SEs on oncogene activation [21]. In TNBC, SEs promote tumor growth, invasion, and drug resistance by driving aberrant overexpression of oncogenes such as MARCO [22]. Whether SEs specifically regulate SPHK1 expression in TNBC has emerged as one of the key focuses of our study.

Given the limitations of Niraparib monotherapy, there is an urgent need to explore novel combination strategies to improve patient survival rates. Artificial intelligence (AI) has been increasingly applied to methods for identifying new drug targets, offering significantly reduced costs and accelerated drug development timelines compared to traditional approaches. AlphaFold has achieved a breakthrough in high-accuracy, end-to-end prediction of biomolecular complex structures, encompassing protein–ligand, protein–DNA/RNA, and protein–post-translational modification interactions, thereby providing near-atomic-resolution models of binding pockets for novel targets that lack experimentally determined crystal structures [23]. Consequently, AlphaFold enables direct generation of virtual screening-ready 3D structures for traditionally “undruggable” targets (e.g., transcription factors, RNA-binding proteins), substantially reducing the cost and time required for experimental structure determination. Furthermore, AlphaFold ‘s ability to predict binding affinities between targets and small molecules offers significant utility for structure-based drug discovery.

Despite the landmark success of PARP inhibitors in breast cancer therapy, the molecular mechanisms underlying resistance—particularly the pro-survival signaling mediated by sphingolipid-metabolic enzyme SPHK1—remain insufficiently understood. The precise transcriptional control of SPHK1 in TNBC, especially whether it is governed by oncogenic SEs, is still unknown. Therefore, this study aims to determine whether TNBC-specific SEs drive SPHK1 expression, thereby establishing a novel SE–SPHK1 axis as a fundamental driver of PARP-inhibitor resistance, and leverage the transformative power of AlphaFold to accelerate the development of next-generation small-molecule inhibitors. We will pre-clinically validate the therapeutic potential of combining these AI-designed small-molecule inhibitors with PARP inhibitors to enhance treatment efficacy in breast cancer.

## 2. Results

### 2.1. Niraparib Exhibits Limited Efficacy and Induces Lipid Metabolic Reprogramming in TNBC Cells

**Figure 1:**
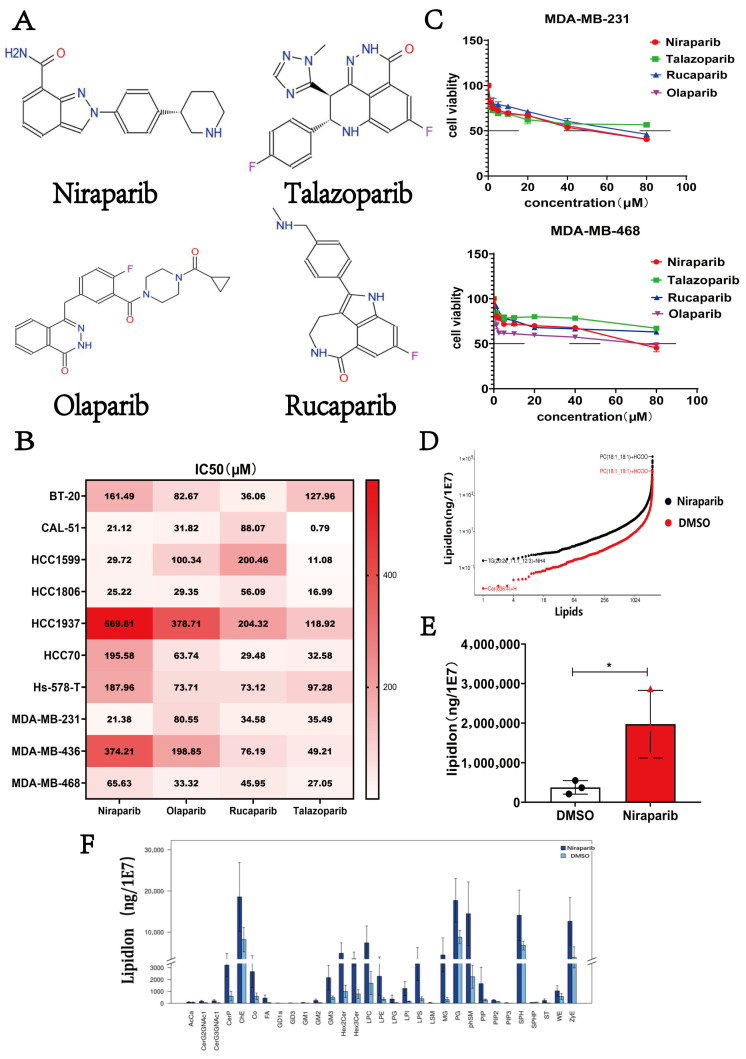

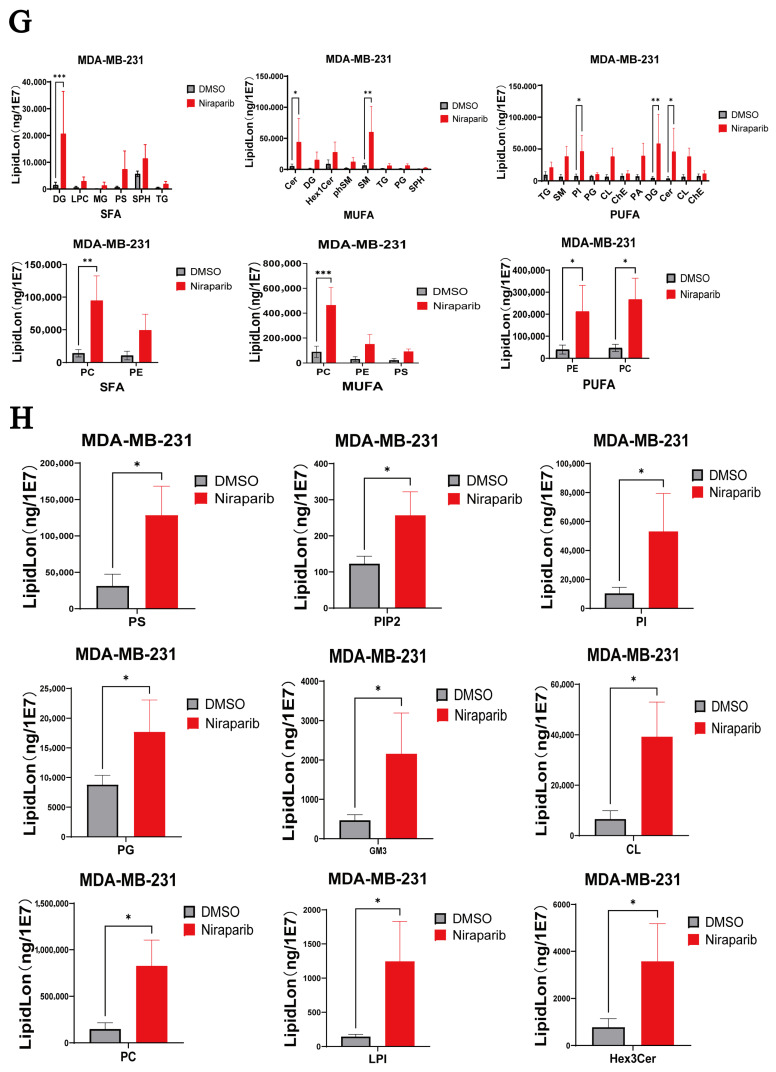
Niraparib causes lipid metabolic reprogramming and is associated with limited efficacy in TNBC cells. (**A**) The chemical structures of the four PARP inhibitors (Niraparib, Talazoparib, Rucaparib, and Olaparib). (**B**) IC50 values of PARPi in TNBC cells from the Genomics of Drug Sensitivity in Cancer database; (**C**) Cell viability of MDA-MB-231 and MDA-MB-468 cell lines treated with Niraparib, Talazoparib, Rucaparib, and Olaparib as measured by MTT assay; The line in the figure represents 50% cell viability; (**D**,**E**) Changes in lipid content in MDA-MB-231 cells treated with 25 μM Niraparib as analyzed by lipid metabolomics sequencing; (**F**) Changes in the relative content of lipid subclasses in cells after Niraparib treatment are displayed using differential bar plots. (The vertical axis, labeled as LipidLon, represents the adduct ion forms of lipid molecules. MK denotes the group treated with Niraparib.) (**G**) Clustering analysis of the content of differential lipids in SFAs, MUFAs, and PUFAs. (**H**) Clustering analysis of lipid molecules with significant differences in total content. Statistical significance between groups is indicated (* *p* < 0.05; ** *p* < 0.01; *** *p* < 0.001).

The chemical structures of four clinically important PARP inhibitors—Niraparib, Talazoparib, Rucaparib, and Olaparib—are shown in Figure 1A. We analyzed data from the Genomics of Drug Sensitivity in Cancer (GDSC) database, which revealed significant heterogeneity in the response of TNBC cell lines to these four PARP inhibitors. This variability underscores the complexity of PARPi sensitivity across different TNBC models. These findings are consistent with clinical observations that the efficacy of PARPi in TNBC is influenced by genomic context, including BRCA1/2 mutation status, homologous recombination deficiency (HRD), and compensatory DNA repair mechanisms (Figure 1B). Therefore, we selected two commonly used TNBC cell lines (MDA-MB-231 and MDA-MB-468) to validate the cytotoxic effects of the four PARP inhibitors. Results showed that all four PARP inhibitors induced a dose-dependent decrease in cell viability in both MDA-MB-231 and MDA-MB-468 cells. However, even at high concentrations (80 μM), all PARP inhibitors only suppressed approximately 50% of cell viability, suggesting intrinsic resistance in these BRCA1 wild-type cell lines. This limited cytotoxicity indicates that PARPi monotherapy may be insufficient for TNBC with functional *BRCA1* and necessitates combination strategies to overcome resistance (Figure 1C). Thus, identifying the mechanisms underlying Niraparib resistance in TNBC is crucial. Given the role of lipid metabolism in cancer, we investigated the impact of Niraparib on lipid metabolism by treating MDA-MB-231 cells with 25 μM Niraparib for 48 h, followed by lipidomic analysis to determine the absolute levels of various lipids in Niraparib-treated versus solvent control groups. As shown in Figure 1D,E, Niraparib significantly increased intracellular lipid accumulation. Since lipid functions are primarily manifested at the subclass level and different subclasses have distinct biological roles, we compared the expression changes in lipid subclasses between the two groups to identify key subclasses regulated by Niraparib (Figure 1F). Given the important influence of lipid unsaturation degree on ferroptosis—saturated fatty acids (SFAs) are generally not directly involved in ferroptosis but may indirectly affect it by increasing membrane rigidity; monounsaturated fatty acids (MUFAs, such as oleic acid) can inhibit ferroptosis by reducing the proportion of polyunsaturated fatty acids (PUFAs) in membranes and decreasing lipid peroxidation; while PUFAs (such as linoleic acid and arachidonic acid) are prone to lipid peroxidation [24,25], generating lipid peroxides that promote ferroptosis [26]—we statistically analyzed differentially expressed lipids (*p* < 0.05) based on their unsaturation. The contents of lipid molecules with the same number of unsaturated bonds were summed, and the total concentrations of saturated, monounsaturated, and polyunsaturated fatty acids were subjected to cluster analysis to compare differences in subclass abundance (Figure 1G). The results showed that both pro-ferroptotic and anti-ferroptotic lipids were upregulated, which explains the limited efficacy of Niraparib monotherapy. Further cluster and statistical analyses indicated that Niraparib induces lipid metabolic reprogramming in TNBC cells (Figure 1H).

### 2.2. Niraparib Induces Dysregulation of Sphingolipid SPH Metabolism and Activates SPHK1

**Figure 2:**
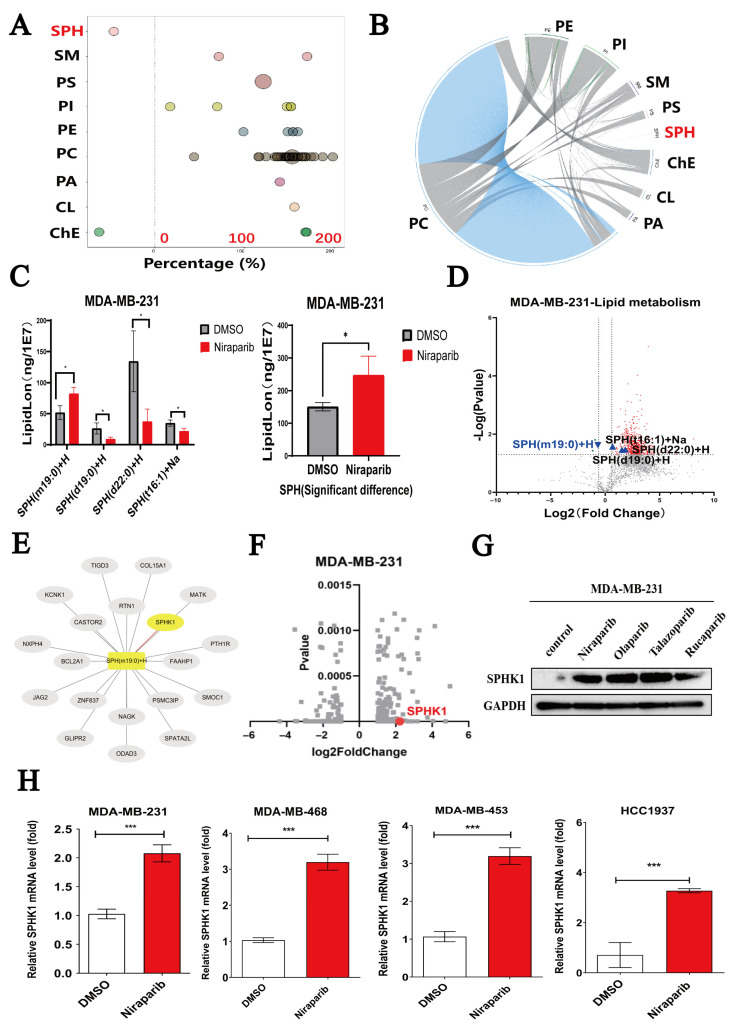
Niraparib promotes SPH accumulation and activates SPHK1 signaling. (**A**,**B**) Relative proportions of different lipid types (SPH, SM, PS, PI, PE, PC, PA, CL, ChE) in MDA-MB-231 cells treated with Niraparib. In the figure A, the bubbles represent significantly different lipid molecules; the y-axis shows the lipid subclasses, distinguished by different colors. The size of each bubble indicates the level of significance: smaller bubbles denote significant differences (0.01 < *p* value < 0.05), while larger bubbles denote highly significant differences (*p* value < 0.01). (**C**) Statistical plots of the total content of four SPH differential lipid molecules and the total content of SPH with *p* < 0.05 in the Niraparib-treated group and the negative control group. (**D**) Volcano plot showing SPH lipid molecules with *p* < 0.05 in the lipid metabolism data. The vertical lines indicate Fold change > 1.5 or Fold change < 0.67; the horizontal line denotes *p*-value < 0.05. (**E**) Correlation network analysis between differential genes and differential sphingolipids. (**F**) Volcano plot showing SPHK1 in the transcriptomics data. (**G**) SPHK1 protein expression in MDA-MB-231 cells treated with PARPi (Niraparib, Olaparib, Talazoparib, Rucaparib) vs. control (Western blot). (**H**) RT-qPCR validation of whether Niraparib significantly upregulates SPHK1 in four TNBC cell lines. Data are presented as the mean ± SEM from three independent experiments. Statistical significance between groups is indicated (* *p* < 0.05; *** *p* < 0.001).

There are numerous classes of lipids, and distinct lipid subclasses play diverse roles in tumor growth. Guided by integrated analysis of lipidomics and transcriptomics data, we focused on the sphingolipid metabolism pathway and its associated gene alterations. Lipid class-specific analysis of Niraparib-treated MDA-MB-231 cells revealed a decrease in sphingosine (SPH) levels (Figure 2A,B), indicating that Niraparib treatment perturbs the sphingolipid metabolic pathway. This pathway is critical because sphingolipids can mediate apoptosis via ceramide or promote cell survival via sphingosine-1-phosphate (S1P). Specifically, four SPH species were significantly altered (*p* < 0.05): SPH(m19:0)+ H, SPH(d19:0)+ H, SPH(d22:0)+ H, and SPH(t16:1)+ H. Notably, the total content of these four differentially expressed SPH molecules was significantly higher than that in the control group (Figure 2C). A volcano plot further illustrates these SPH changes, with downregulated SPH lipids marked in blue and upregulated ones in black (Figure 2D). Collectively, these results demonstrate that Niraparib modulates SPH metabolism. To systematically identify gene changes associated with sphingolipid metabolic dysregulation in TNBC, we further integrated lipidomics and transcriptomics data. A Pearson correlation network analysis (|r| ≥ 0.5, *p* < 0.05) revealed an interaction network centered on SPH, linking its changes to SPHK1 (Figure 2E). Transcriptomic results showed that Niraparib significantly upregulates SPHK1 gene expression (Figure 2F). This upregulation was confirmed at the protein level: all four tested PARP inhibitors (Niraparib, Olaparib, Talazoparib, and Rucaparib) markedly increased SPHK1 protein levels in MDA-MB-231 cells (Figure 2G). RT-qPCR further validated that Niraparib promotes SPHK1 transcription in TNBC cells (Figure 2H). Therefore, Niraparib upregulates the key metabolic enzyme SPHK1 and induces sphingolipid metabolic disorder in TNBC cells. Analysis of publicly available breast-cancer datasets revealed that SPHK1 expression is markedly elevated in TNBC patient samples and that high SPHK1 levels are significantly associated with poor prognosis (Figure S1A–C).

### 2.3. Niraparib Potentiates Ferroptosis in TNBC Cells by Targeting SPHK1

**Figure 3:**
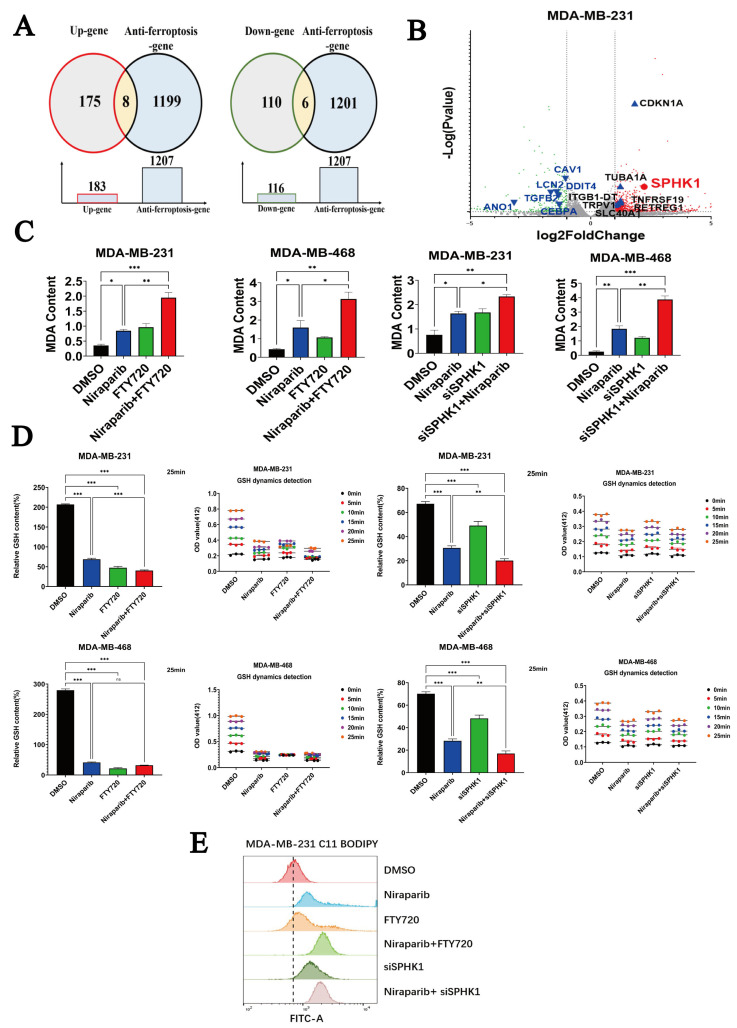
Niraparib sensitizes TNBC cells to ferroptosis by inhibiting SPHK1 activity. (**A**) Venn diagram showing the intersection of differentially expressed genes from lipid metabolism transcriptomic data and anti-ferroptosis-related genes from the GeneCards database. (**B**) Volcano plot of differentially expressed genes generated using GraphPad Prism software (version 10.1.2), with the intersecting genes highlighted. The dashed lines in the figure represent Fold change = 2 and Fold change = −2. (**C**) Measurement of MDA levels in four groups of cells after pharmacological inhibition of SPHK1 (FTY720) or siRNA-mediated knockdown of SPHK1. (**D**) Measurement of GSH levels in four groups of cells after pharmacological inhibition of SPHK1 (FTY720) or siRNA-mediated knockdown of SPHK1. Data are presented as the mean ± SEM from three independent experiments. (**E**) The level of lipid peroxides was quantified by C11 BODIPY 581/591 labeling and flow cytometry. Statistical significance between groups is indicated (* *p* < 0.05; ** *p* < 0.01; *** *p* < 0.001).

Since lipid peroxidation is closely associated with ferroptosis and the cytotoxic effect of Niraparib is limited, we hypothesized that Niraparib might activate anti-ferroptosis mechanisms. To test this hypothesis, we performed a combined analysis of the lipid metabolism transcriptome data and the GeneCards database. From GeneCards, we identified 1207 genes related to anti-ferroptosis and intersected them with the differentially expressed genes (upregulated or downregulated) from the transcriptome. The results showed that 8 genes overlapped with the upregulated gene set and 6 genes overlapped with the downregulated gene set (Figure 3A). These intersecting genes are annotated on the volcano plot of the transcriptomic differentially expressed genes: the x-axis represents the log2-transformed fold change in gene expression, and the y-axis represents the negative log10-transformed *p* value. Gray dots represent non-differentially expressed genes, red dots represent upregulated genes, and green dots represent downregulated genes. Blue upward triangles on the right side mark the 8 anti-ferroptosis-related genes found among the upregulated genes (*SPHK1*, *CDKN1A*, *TNFRSF19*, *RETREG1*, *ITGB1-DT*, *TUBA1A*, *SLC40A1*, *TRPV1*); blue downward triangles on the left side mark the 6 anti-ferroptosis-related genes found among the downregulated genes (*ANO1*, *LCN2*, *DDIT4*, *TGFβ2*, *CEBPA*, *CAV1*) (Figure 3B). Strikingly, we found that Niraparib induced the upregulation of SPHK1, a sphingolipid metabolic enzyme gene, and database results suggested that this gene may trigger an anti-ferroptosis mechanism.

MDA and GSH are two critical biomarkers in ferroptosis, reflecting the level of lipid peroxidation and cellular antioxidant capacity, respectively, and are closely linked to the occurrence of ferroptosis. MDA is a terminal product of lipid peroxidation, and its elevated level directly indicates ferroptosis induction; GSH is a key intracellular antioxidant that inhibits lipid peroxidation and ferroptosis by maintaining GPX4 activity [27,28]. Therefore, we further investigated the impact of targeting SPHK1 on Niraparib-induced ferroptosis. FTY720 (Fingolimod), an immunomodulatory drug initially used for treating multiple sclerosis (MS), can influence sphingolipid metabolism and cell survival by inhibiting SPHK1 activity [29]. Experimental results showed that Niraparib induced an increase in intracellular MDA levels and a decrease in GSH levels; however, pharmacological inhibition (FTY720) or knockdown of SPHK1 potentiated the Niraparib-induced increase in MDA and decrease in GSH. (Figure 3C,D). Similarly, we employed C11 BODIPY 581/591 flow-cytometry to quantify lipid peroxidation—the direct hallmark of ferroptosis. The data show that Niraparib alone triggers a moderate level of ferroptosis, whereas either SPHK1 knock-down or pharmacological inhibition with FTY720 markedly sensitizes cells to Niraparib-induced ferroptosis.

### 2.4. Niraparib-Induced Activation of PI3K-AKT and MAPK Pathways Is Significantly Reversed by SPHK1 Knockdown

**Figure 4:**
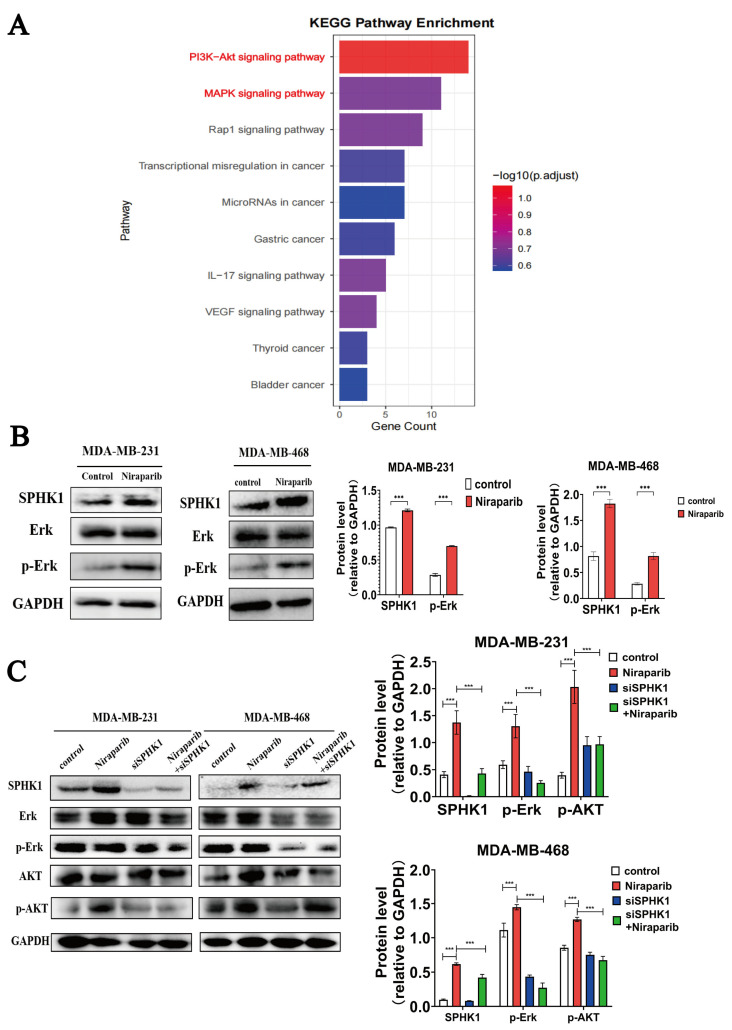
Niraparib-induced activation of PI3K-AKT and MAPK pathways is rescued by SPHK1 knockdown. (**A**) KEGG pathway enrichment analysis of transcriptomic sequencing data from MDA-MB-231 cells treated with Niraparib. (**B**) Western blot analysis of the expression levels of Erk and p-Erk pathway proteins in TNBC cell lines (MDA-MB-231 and MDA-MB-468) treated with Niraparib. (**C**) Western blot validation showing that knockdown of SPHK1 reverses the Niraparib-induced upregulation of SPHK1 and the activation of the PI3K/AKT and MAPK signaling pathways in TNBC cells. Statistical significance between groups is indicated (*** *p* < 0.001).

SPHK1 activates the PI3K-AKT and MAPK pathways by generating sphingosine-1-phosphate (S1P), thereby regulating cell survival, proliferation, metabolism, and inflammatory responses; conversely, the PI3K-AKT and MAPK pathways can provide feedback regulation on SPHK1, forming a positive feedback loop that further amplifies signal transduction [30]. To investigate the signaling pathways affected by Niraparib in TNBC cells, we performed KEGG enrichment analysis on transcriptomic sequencing data. The results indicated that both the PI3K/AKT and MAPK signaling pathways were among the most significantly altered pathways (Figure 4A). Previous work from our group has confirmed that treatment of TNBC cells with Niraparib for 48 h activates the PI3K-AKT growth signaling pathway; we therefore further verified that Niraparib also activates the MAPK growth-related signaling pathway, contributing to the development of drug resistance (Figure 4B). Knockdown of SPHK1 reversed the Niraparib-induced upregulation of SPHK1 expression and inhibited the activation of both PI3K/AKT and MAPK signaling pathways by Niraparib in TNBC cells (Figure 4C). In summary, Niraparib activates the PI3K/AKT and MAPK signaling pathways in TNBC cells via SPHK1, and knockdown of SPHK1 significantly suppresses the activity of these pathways.

### 2.5. SPHK1 Is Identified as a Super-Enhancer-Driven Gene in TNBC

SPHK1 is a key target that sensitizes TNBC cells to Niraparib. However, existing SPHK1 inhibitors can only inhibit its function without altering the protein content of SPHK1, which often leads to drug resistance due to point mutations. Therefore, how to fundamentally eliminate Niraparib resistance induced by SPHK1 upregulation has become an important issue that needs to be resolved urgently. Given this, it is necessary to explore how Niraparib affects the expression of SPHK1 in order to reduce its expression from the source. Considering that SEs can drive the expression of cell identity genes [31], do TNBC cells (one of the most difficult types of breast cancer cells to treat) have unique SEs? In addition, is there a connection between Niraparib -induced upregulation of SPHK1 and SEs in TNBC?

**Figure 5:**
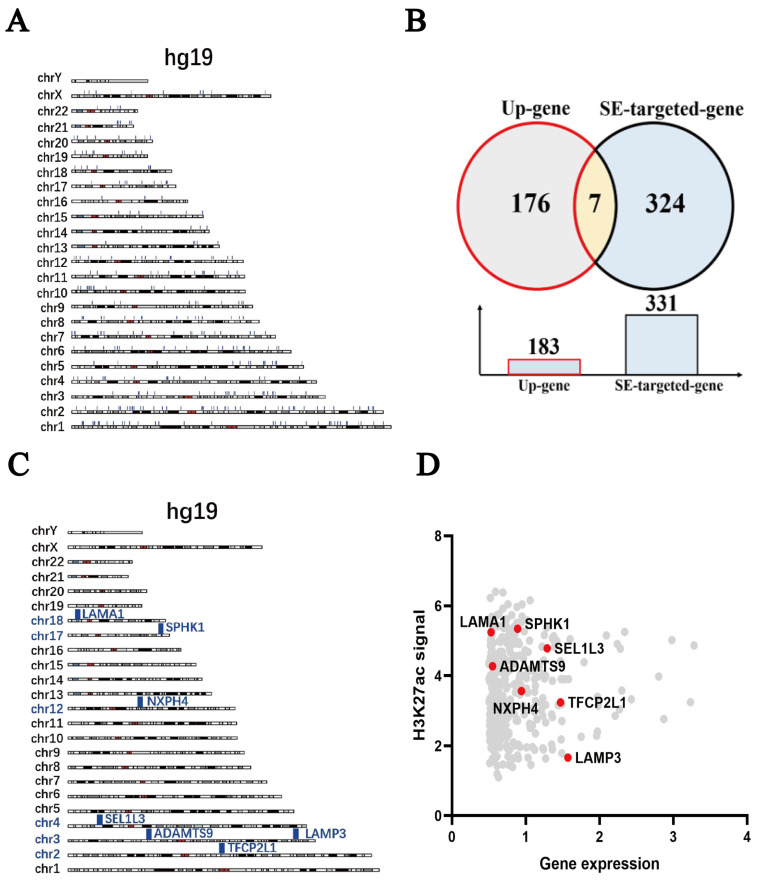

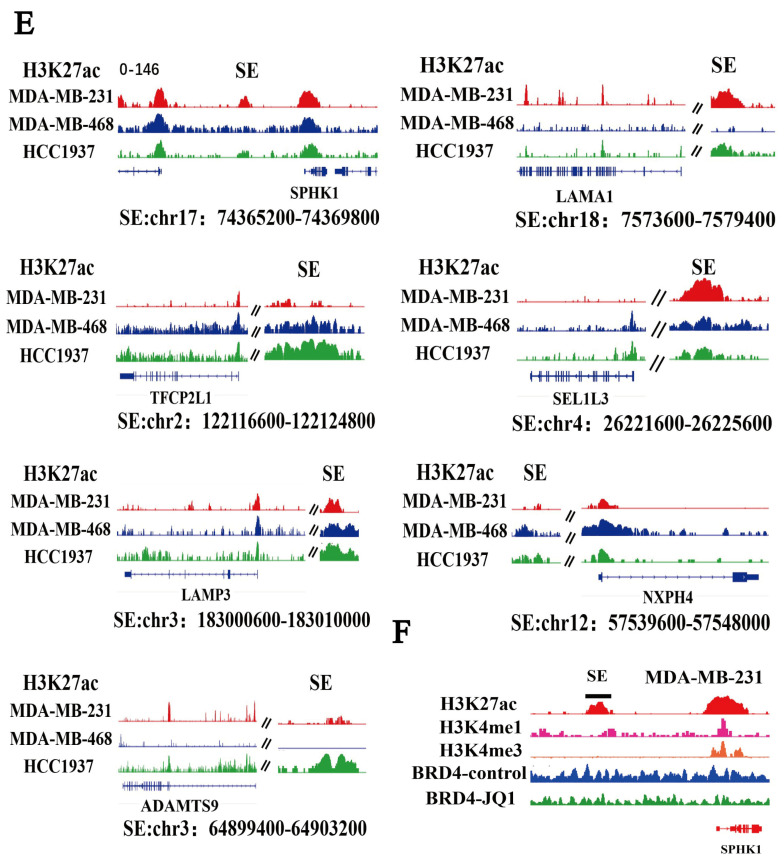

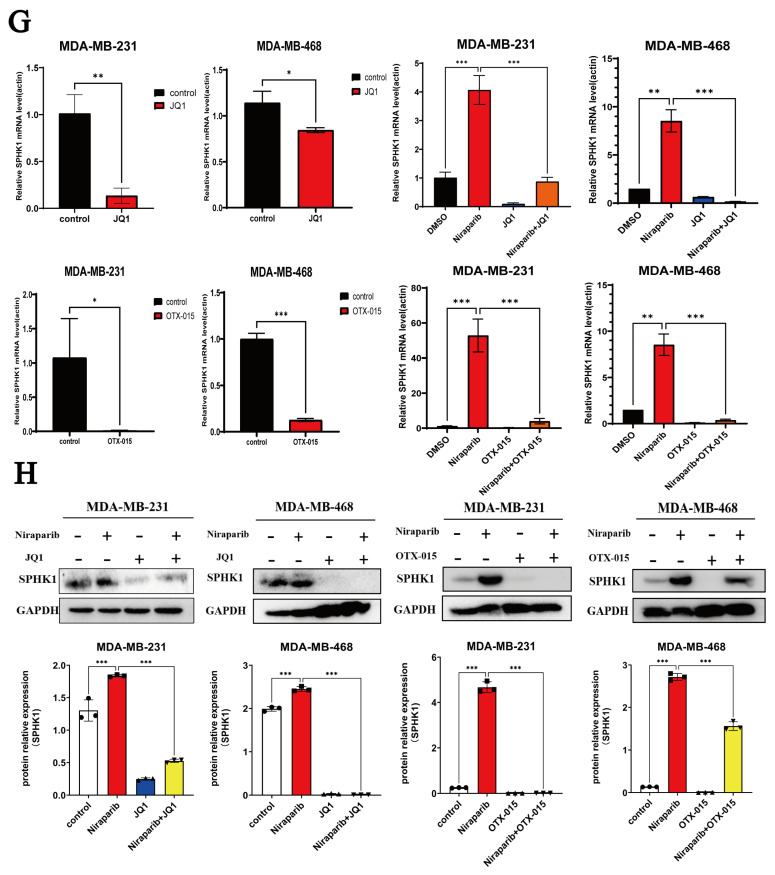
The transcriptional regulation of *SPHK1* is governed by a super-enhancer in TNBC. (**A**) Distribution map of H3K27ac signals for 331 TNBC-specific SEs across the genome, drawn using the MicroSignal website; (**B**) Venn diagram of target genes of 331 TNBC-specific SEs and differentially expressed genes in the transcriptome; (**C**) Distribution map of H3K27ac signals for 7 genes across the genome, drawn using the MicroSignal website; (**D**) Graph of H3K27ac signals for 331 TNBC-specific SE target genes, drawn using GraphPad Prism software (version 10.1.2); (**E**) IGV display of H3K27ac signal peaks for 7 gene-SEs in 3 TNBC cell lines; (**F**) IGV visualization of H3K27ac/H3K4me1/H3K4me3/BRD4 signals in MDA-MB-231 cells; (**G**) RT-qPCR validation of SPHK1 mRNA levels in TNBC cells treated with 4 μM JQ1/OTX-015 for 24 h, and verification of whether BRD4 inhibitors can reverse the upregulation of SPHK1 expression caused by Niraparib; (**H**) Western blot validation of whether BRD4 inhibitors can reverse the upregulation of SPHK1 protein expression caused by Niraparib. Data are presented as the mean ± SEM from three independent experiments. Statistical significance between groups is indicated (* *p* < 0.05; ** *p* < 0.01; *** *p* < 0.001).

Huang, H. and colleagues used a “multi-omics analysis” approach to identify SEs unique to TNBC and obtained 331 SE target genes specific to TNBC [32]. Figure 5A shows the genome-wide distribution of H3K27ac signals of these 331 TNBC-specific SEs. By cross-analyzing these 331 SE-regulated target genes with differentially expressed genes in MDA-MB-231 cells treated with Niraparib, a Venn diagram (Figure 5B) was generated, revealing seven overlapping genes: *SPHK1*, *LAMA1*, *SEL1L3*, *ADAMTS9*, *NXPH4*, *TFCP2L1*, and *LAMP3*. Figure 5C shows the genome-wide distribution of H3K27ac signals of these seven genes. Using GraphPad Prism software, a plot of H3K27ac signals for the 331 SE target genes was created, with the x-axis representing the log_2_ (FoldChange) value of each gene and the y-axis representing the H3K27ac signal value of Gene-SE. In this figure, the seven overlapping genes are marked in red, and *SPHK1*-SE has the strongest H3K27ac signal (Figure 5D). We retrieved H3K27ac-ChIP-seq data from the GEO database for different cell lines (MDA-MB-231, MDA-MB-468, HCC1937) and further analyzed the H3K27ac peaks for the seven selected genes (*SPHK1*, *LAMA1*, *SEL1L3*, *ADAMTS9*, *NXPH4*, *TFCP2L1*, *LAMP3*). We found that the SE regulating *SPHK1* was the most active (Figure 5E). We also retrieved H3K27ac/H3K4me1/H3K4me3-ChIP-seq data and BRD4/BRD4-JQ1-ChIP-Seq data for MDA-MB-231 from the GEO database. IGV visualization showed that in the *SPHK1*-SE region of MDA-MB-231 cells, H3K27ac and H3K4me1 expression was high, while H3K4me3 expression was low. BRD4 was highly enriched in the *SPHK1*-SE region. The BRD4 inhibitor JQ1 effectively reduced BRD4 expression (Figure 5F). These findings indicate that TNBC-specific SEs can regulate *SPHK1* expression.

JQ1 and OTX-015, as BET inhibitors, can effectively regulate the activity of super-enhancers by inhibiting the activity of BRD4 and other proteins and are commonly used to study the activity of super-enhancers [33,34]. In TNBC cells treated with 4 μM BRD4 inhibitors (JQ1/OTX-015) for 24 h, RT-qPCR experiments confirmed the decreased mRNA level of the SPHK1 gene. Further RT-qPCR experiments confirmed that BRD4 inhibitors can reverse the upregulation of SPHK1 expression induced by Niraparib (Figure 5G). Similarly, we also validated this at the protein level. The results showed that BRD4 inhibitors can reverse the upregulation of SPHK1 protein expression induced by Niraparib (Figure 5H). In summary, these results provide strong evidence that SPHK1 expression in TNBC cells is regulated by SE.

### 2.6. Niraparib-Induced SPHK1 Activation Is Significantly Reversed by Targeted Inhibition of Super-Enhancer Activity

**Figure 6:**
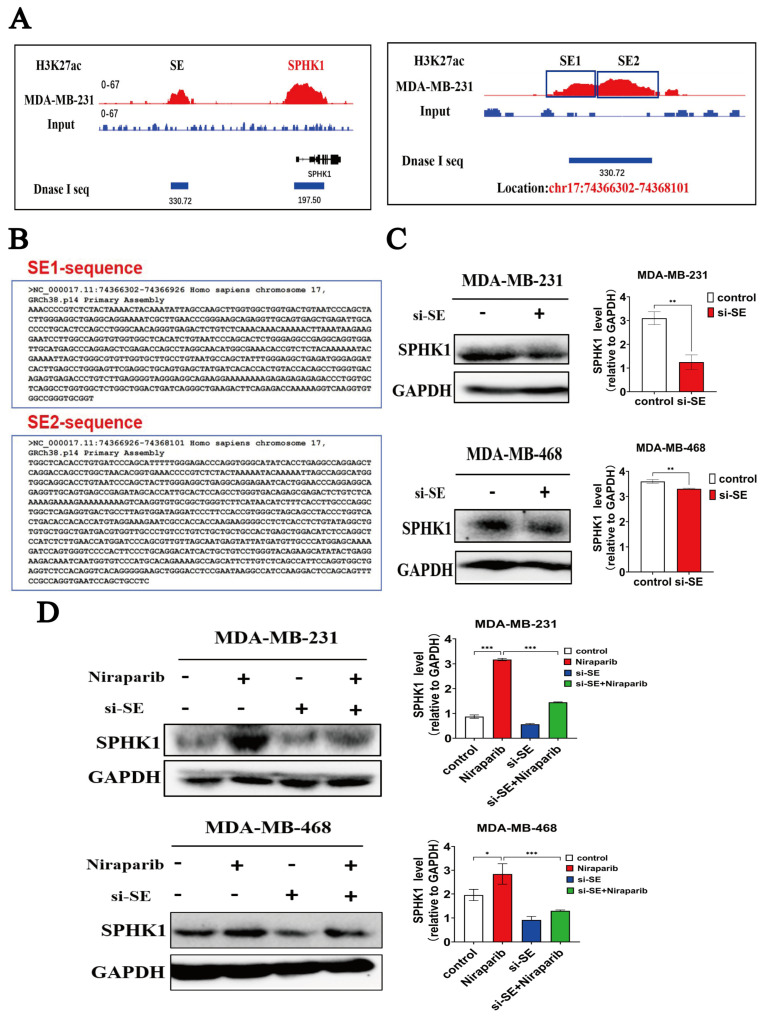
Targeted inhibition of super-enhancer activity reverses Niraparib-induced SPHK1 activation. (**A**) IGV software visualization of the SE peak corresponding to SPHK1 and the genomic location of the SE sequence. (**B**) NCBI database mapping of the sequences corresponding to SPHK1-SE1 and SE2. (**C**) Western blot analysis to determine whether the introduction of siRNA-SE in TNBC cells leads to downregulation of SPHK1. (**D**) Western blot analysis to determine whether the introduction of siRNA-SE in TNBC cells reverses the Niraparib-induced upregulation of SPHK1. Statistical significance between groups is indicated (* *p* < 0.05; ** *p* < 0.01; *** *p* < 0.001).

An important question warranting further investigation is whether targeted inhibition of super-enhancer activity downregulates SPHK1 expression and reverses its upregulation induced by Niraparib. We obtained H3K27ac-ChIP-seq and DNase I-Seq data of MDA-MB-231 cells from the GEO database and visualized the super-enhancer (SE) peaks corresponding to SPHK1 using IGV software(version 2.17.4). The results revealed that this SE region is located at chr17: 74,366,302-74,368,101. Based on peak intensity, it was further divided into SE1 and SE2 (Figure 6A). Subsequent analysis using the NCBI database confirmed the genomic coordinates of SE1 as chr17:74,366,302-74,366,926 and SE2 as chr17:74,366,926-74,368,101 (Figure 6B). We designed siRNA-SE targeting the SPHK1-SE2 sequence, which exhibited stronger signal intensity. Western Blot analysis demonstrated that transfection with siRNA-SE in TNBC cells suppressed SPHK1 expression and reversed the Niraparib-induced upregulation of SPHK1 (Figure 6C,D).

### 2.7. Niraparib Promotes SPHK1 Transcriptional Activation Through SP1 Recruitment and Super-Enhancer Engagement

**Figure 7:**
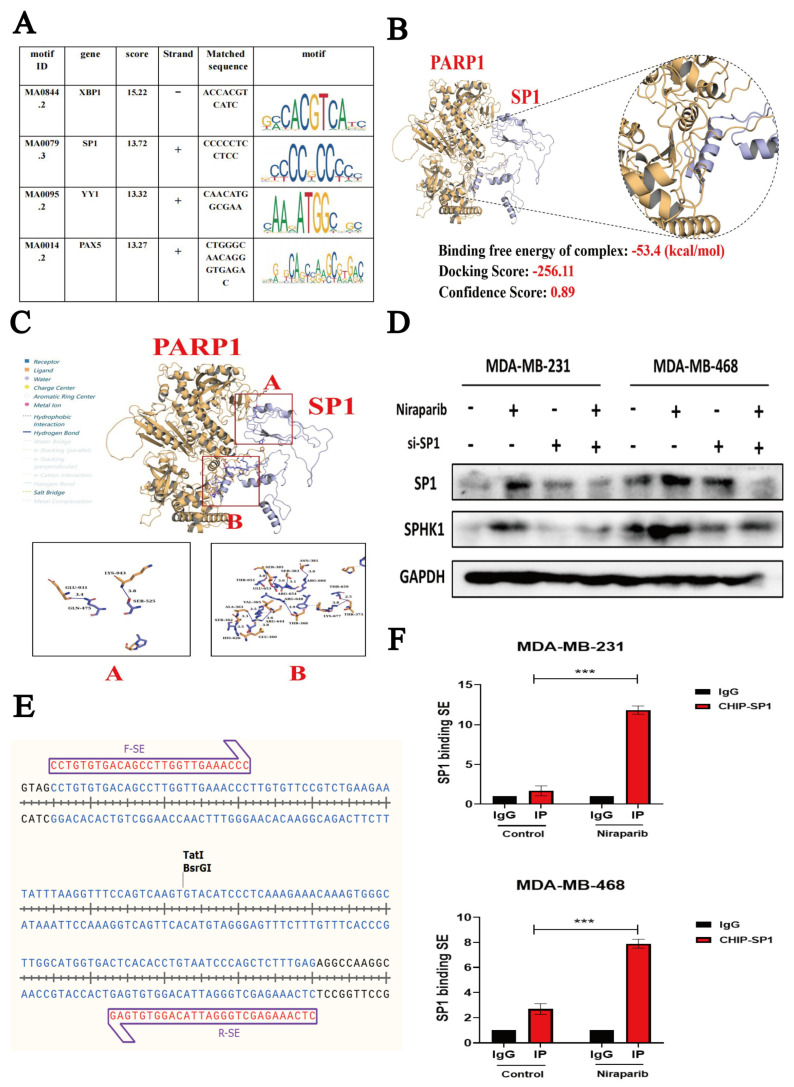

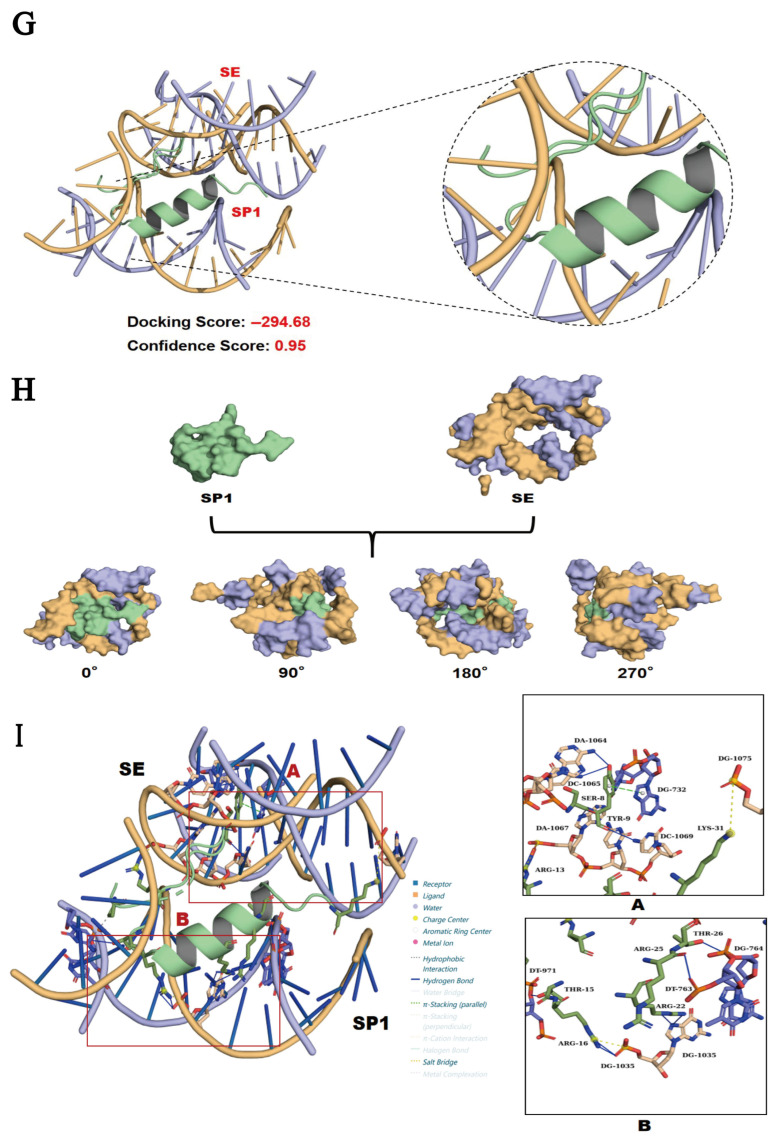

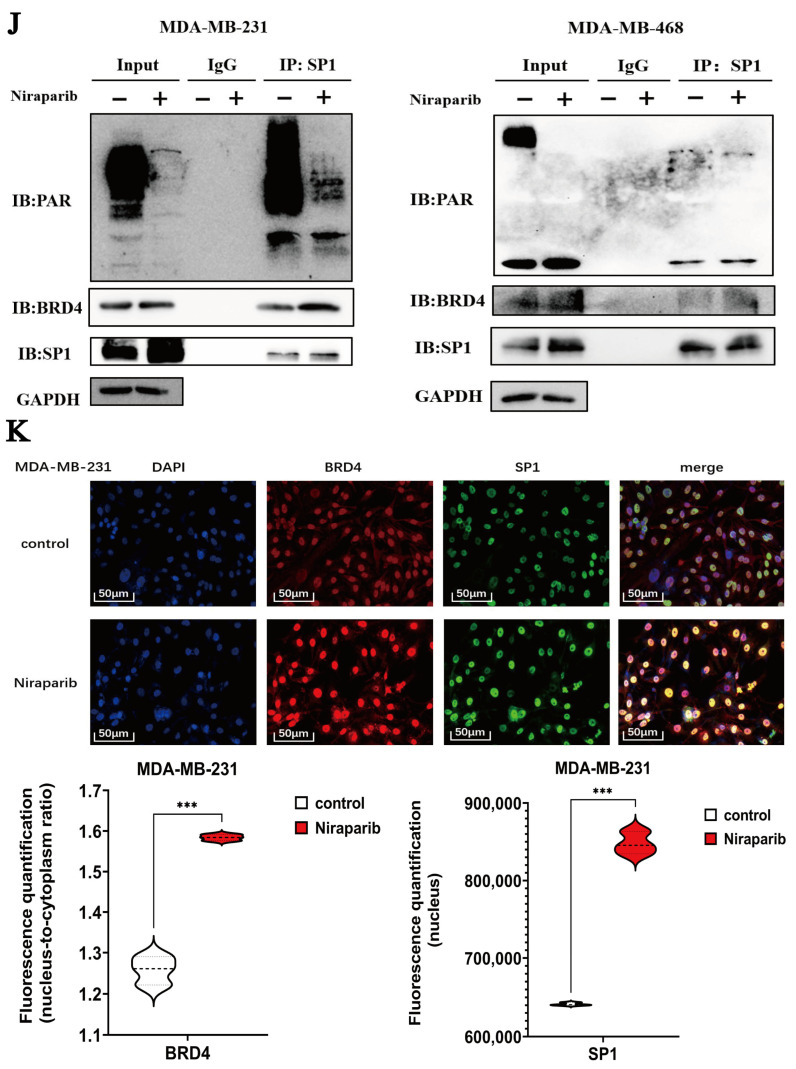
Niraparib facilitates SP1-mediated super-enhancer activation and subsequent SPHK1 transcription. (**A**) Prediction of transcription factor binding sites on *SPHK1*-SE using the PROMO and JASPAR websites. Top four transcription factors ranked by prediction scores. (**B**,**C**) PyMOL visualization of the docking model between the transcription factor SP1 and the PARP1 protein. (**D**) Western blot analysis to determine whether the introduction of siRNA-SP1 in TNBC cells reverses the Niraparib-induced upregulation of SPHK1. (**E**) Design of specific primers (F-SE/R-SE) targeting the blue SE sequence using SnapGene software. (**F**) ChIP-qPCR analysis to determine whether Niraparib promotes the binding of the SP1 transcription factor to the SE sequence. (**G**–**I**) The docking results of SP1 protein with SE sequence. Green indicates SP1; all other regions represent SE. (**J**) Co-IP assay to detect whether Niraparib inhibits the PARylation of SP1 in TNBC cells. (**K**) Immunofluorescence assay to determine whether Niraparib promotes the nuclear translocation of SP1 and BRD4 and enhances the fluorescence signals of these two proteins. The BRD4 fluorescence signal was quantified as the nuclear-to-cytoplasmic intensity ratio. The SP1 fluorescence signal was quantified based on its intensity within the nucleus. Statistical significance between groups is indicated (*** *p* < 0.001).

We aimed to further investigate which TFs bind to this SE. To this end, we used the PROMO website to predict TFs that bind to SPHK1-SE, with a mismatch tolerance set at 1%, and employed the JASPAR website to predict transcription factor binding sites on SPHK1-SE. Based on the ranking of prediction scores, we identified the top four transcription factors (Figure 7A). We used PLIP to analyze the interaction between SP1 and PARP1. Yellow represents PARP1, and purple represents SP1. The free binding energy is −53.4 kcal/mol. The docking score of SP1 and PARP1 is −256.11, with a confidence score of 0.89, indicating that the binding of the two proteins in this form is highly credible (Figure 7B,C). The results indicated that SP1 can bind to both SPHK1-SE and interact with the PARP1 protein. According to the JASPAR website predictions, SP1 has a high binding score on the positive strand and exhibits a larger binding free energy with the PARP1 protein. Studies have shown that SP1 can directly bind to specific GC-rich sites in the promoter region of the *SPHK1* gene, enhancing its transcriptional activity [35]. Additionally, research has demonstrated that SP1 can activate the expression of RGS20 through a super-enhancer to promote the progression of lung adenocarcinoma [36]. Therefore, SP1 may play a dominant role in the activity of *SPHK1*-SE, and we prioritized SP1 as the primary focus of our research.

We validated that the introduction of siRNA-SP1 in TNBC cells reversed the Niraparib-induced upregulation of SPHK1 (Figure 7D). To further investigate whether Niraparib promotes the binding of the SP1 transcription factor to the SE sequence, we designed specific primers (F-SE/R-SE) targeting the blue SE sequence shown in the figure using SnapGene software (version 7.1.2) (Figure 7E). ChIP-qPCR experiments demonstrated that Niraparib promotes the binding of the SP1 transcription factor to the SE sequence in TNBC cells (Figure 7F). Further, we validated the binding affinity of SP1 to SE sequences through molecular docking. Using AlphaFold3, we modeled the SE sequences and selected the highest-confidence model based on the pLDDT score as the experimental model. Protein-DNA docking was performed using the HDOCKlite v1.1 local server. The results showed a minimum binding energy docking score of −294.68 and a confidence of 0.95, indicating a valid protein–DNA binding conformation (Figure 7G). The binding process was visualized as a surface representation (Figure 7H). We further analyzed the binding mode between SP1 and SE: green represents SP1, yellow represents SE chain A, and purple represents SE chain B. The interaction formed 11 hydrogen bonds (within 4.1 Å), 2 salt bridges, and 1 Pi-Pi stacking interaction, as shown in Figure 7I-A. Specifically, as illustrated in Figure 7I-B, hydrogen bonds were formed between THR-26 of SP1 and G-764 of DNA chain B, ARG-25 of SP1 and T-763 of SE chain B, ARG-22 of SP1 and G-1035 of SE chain A, and ARG-16 of SP1 and G-1035 of SE chain A (with an additional salt bridge). Additionally, a hydrogen bond was formed between T-791 of SE chain B and THR-15 of SP1. Multiple hydrophobic interactions (gray dashed lines) were also observed (Figure 7I).

Previous studies have indicated that transcription factor SP1 can be poly(ADP-ribosyl)ated (PARylated) by PARP1, directly influencing its ability to bind DNA [37,38]. To investigate whether Niraparib enhances the transcriptional activity of SP1 by inhibiting its PARylation, co-immunoprecipitation (Co-IP) assays demonstrated that Niraparib suppresses PARylation of SP1 in TNBC cells (Figure 7J). Immunofluorescence results further revealed that Niraparib promotes the translocation of both SP1 and BRD4 into the nucleus, with a significant increase in their fluorescence signals. Quantitative analysis was performed on the fluorescence signals of BRD4 and SP1. For BRD4, the nuclear-to-cytoplasmic intensity ratio was measured. For SP1, which was predominantly localized in the nucleus, the fluorescence intensity within the nucleus was quantified. The results were consistent with the aforementioned conclusion (Figure 7K). In summary, Niraparib inhibits PARylation of SP1 and facilitates its association with super-enhancers, thereby enhancing the transcriptional activity of SP1 on the *SPHK1* gene.

### 2.8. Niraparib Efficacy Is Significantly Enhanced by Combination Therapy with SP1 and SPHK1 Inhibitors

**Figure 8:**
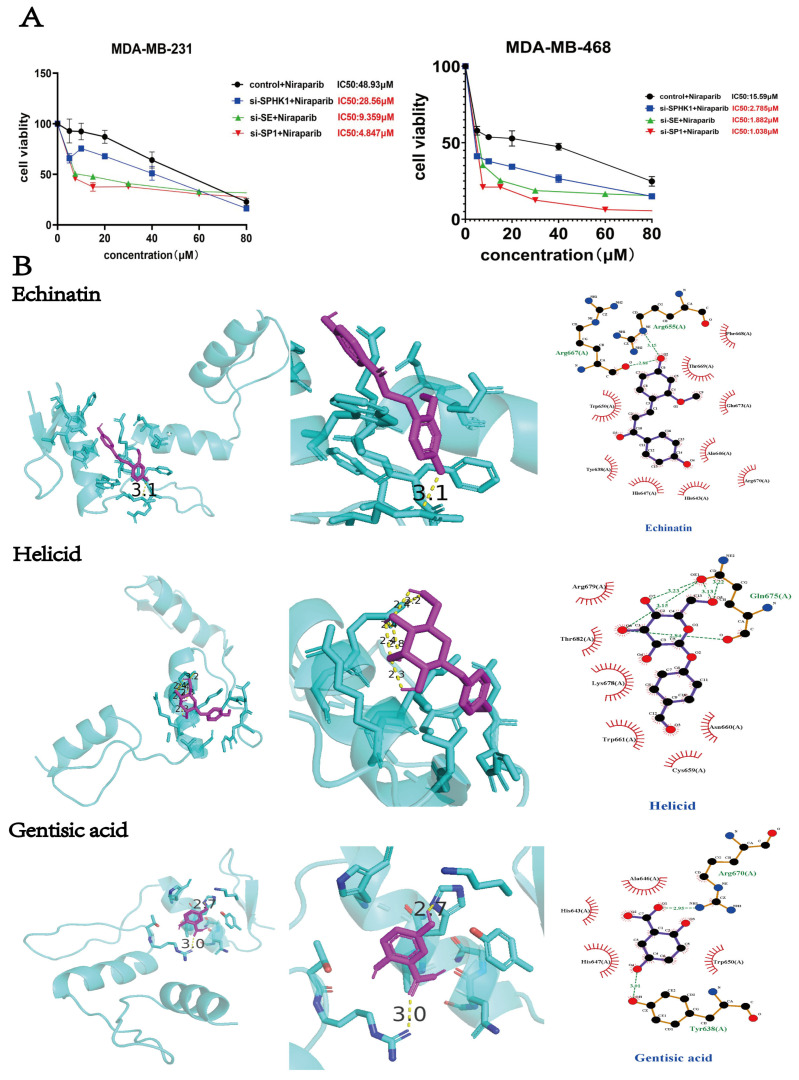

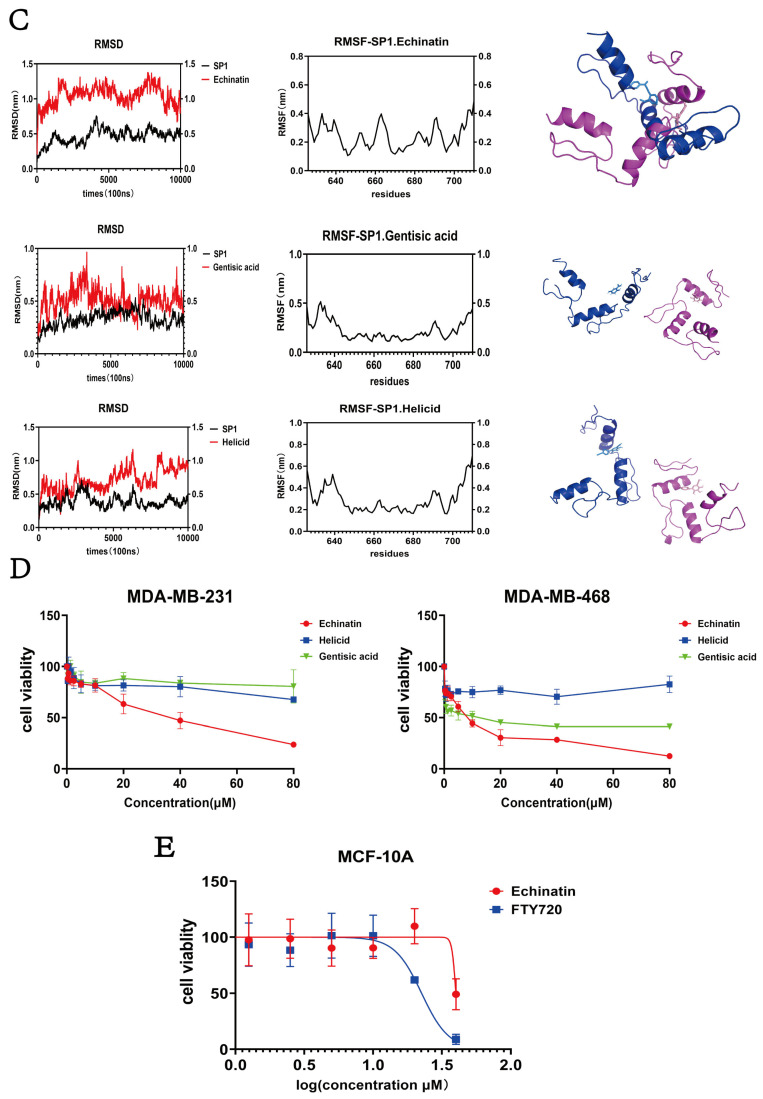

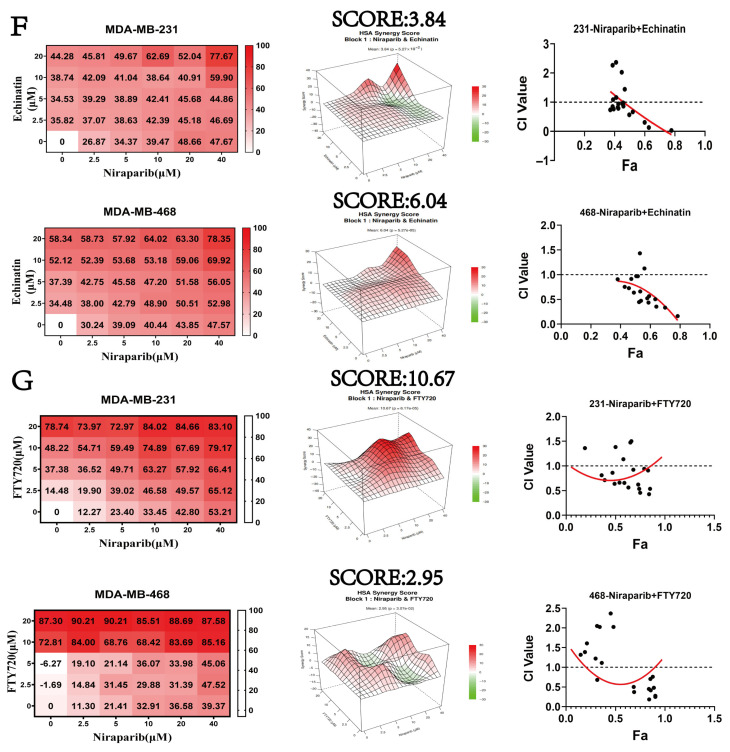

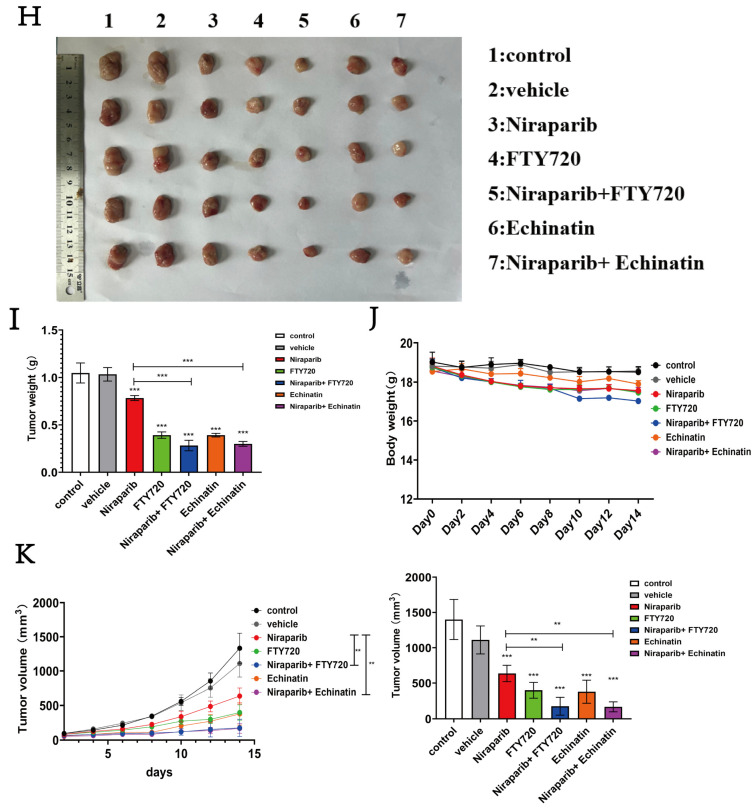
Combined inhibition of SP1 and SPHK1 markedly potentiates the anti-tumor efficacy of Niraparib. (**A**) MTT assays were performed to evaluate the cytotoxic effects of Niraparib in TNBC cells following 24 h treatment with siRNA targeting SPHK1, SE, or SP1. (**B**) Molecular docking diagrams illustrating the binding modes of the three candidate compounds to SP1 were generated using PyMOL (Version 2.5.5) and LigPlot+ (Version 2.2.9); (**C**) The binding stability of these complexes was further assessed via molecular dynamics simulations using GROMACS (Version 2023.2); (**D**) Dose-dependent cytotoxicity of the three compounds against TNBC cells was determined by MTT assay after 72 h of exposure; (**E**) The toxicity of Echinatin on normal MCF-10A cells was evaluated using MTT assay following 72 h of treatment. (**F**,**G**) Following 72 h of treatment with the combination of Niraparib and Echinatin or FTY720 in two TNBC cell lines, cell inhibition rates were measured using the MTT assay. The synergistic effects of the drug combinations were evaluated with SynergyFinder, employing the HSA model for quantitative scoring. Subsequently, the combination index (CI) curves were simulated using CompuSyn software (version 1.0.1) to further assess drug–drug interactions. In the CI-Fa curve plot, the dashed line denotes CI = 1. (**H**) Evaluation of the anti-tumor activity of combination regimens in a 4T1 tumor-bearing mouse model. Representative image of tumors from each group of mice (*n* = 5). (**I**) Quantitative analysis of tumor weight in each mouse group. (**J**) Body weight statistics for each group of mice. (**K**) Tumor growth curves and quantitative analysis of tumor volume for each group of mice. Statistical significance between groups is indicated (** *p* < 0.01; *** *p* < 0.001).

Having established the critical role of SP1 within the super-enhancer and recognizing that enhanced SE activity promotes SPHK1 expression—while disruption of the SE complex can severely disrupt transcriptional processes through multiple mechanisms [39]—we employed siRNA to target components of the SP1/SE/SPHK1 axis to determine which intervention most significantly enhances the efficacy of PARPi in eliminating TNBC cells. After transfecting two TNBC cell lines with various siRNAs for 24 h, we assessed cell viability via MTT assay under Niraparib treatment. As shown, all three siRNAs reduced the IC50 of Niraparib; however, siRNA-SP1 produced the most pronounced effect, lowering the IC50 by approximately 10-fold in TNBC cells, underscoring the pivotal role of SP1 in sustaining SE activity (Figure 8A). Consequently, we focused on SP1 as a therapeutic target. Although several SP1 inhibitors have been explored preclinically, development remains in the early stages, with ongoing efforts to identify agents offering greater efficacy and reduced toxicity. Among these, Mithramycin A (also known as Plicamycin), an antitumor antibiotic derived from Streptomyces plicatus with additional neuroprotective properties, is the most extensively studied. It functions as an inhibitor of both DNA/RNA polymerases and the transcription factor SP1 [40]. We initially selected this compound to evaluate its potency and toxicity in TNBC models. Results indicated that Mithramycin A exhibited substantial toxicity, rendering it unsuitable for further therapeutic development (Figure S2).

The rapid advancement of AI technology has established a comprehensive framework for its application in drug discovery, covering the entire process from “target identification → virtual screening → preclinical validation.” This integrated approach is significantly shortening development timelines, reducing costs, and improving success rates. Having identified SP1 as a critical target, we employed the “DrugRep [41]-DrugFlow [42]-AlphaFold” pipeline to perform virtual screening against a library of traditional Chinese medicine compounds. Within the DrugFlow workflow, an initial high-throughput screening was conducted using “KarmaScore,” followed by a more refined screening with “RTMScore,” a binding affinity scoring function where higher values indicate stronger predicted binding. The top 30 ranked compounds were selected for further evaluation using both AutoDock (version 4.2.6) and AlphaFold (DeepMind Technologies Limited., London, UK) for scoring. AlphaFold predictions were interpreted based on ipTM scores: ≥0.8 (high confidence), 0.6–0.8 (medium confidence), and <0.6 (low confidence). Subsequently, pharmacokinetic and toxicity predictions were performed. LogP, the logarithm of the partition coefficient of a compound in an n-octanol/water system, was used as a key measure of lipophilicity. Compounds with LogP > 5 were excluded due to high lipophilicity, which is associated with poor solubility, increased metabolic burden, potential hERG inhibition, and risk of systemic accumulation; hence, compound number10 was eliminated. Higher LogP values indicate greater lipophilicity (“oil-like”), while lower values suggest higher hydrophilicity (“water-like”). Additionally, we evaluated each compound’s potential carcinogenicity, hepatotoxicity index, acute oral toxicity in rats, and cardiotoxicity. Values exceeding 0.5 were considered indicative of significant toxicity and are highlighted in red in the Table S1 (Supplementary Materials). Based on these comprehensive filters, three compounds—Echinatin, Helicid, and Gentisic acid—were identified as meeting all selection criteria.

We present both 2D and 3D docking conformation diagrams of these three compounds with SP1 (Figure 8B). To further evaluate the binding stability, molecular dynamics (MD) simulations were performed. The Root Mean Square Deviation (RMSD) was used to measure the overall structural deviation of the system relative to the reference conformation (typically the energy-minimized initial structure). An increase in RMSD during the first 1–2 ns of the trajectory indicates a “warming-up” phase of the system, followed by stabilization when the system reaches equilibrium. A continuous increase in RMSD beyond 3–4 Å suggests instability or issues with the initial structure. The Root Mean Square Fluctuation (RMSF) reflects the local flexibility of individual atoms or residues during the equilibrium phase. The results demonstrate that the binding conformations of all three compounds with SP1 remained stable. The rightmost panel illustrates the conformational changes from the first to the last frame over the 100 ns MD simulation (Figure 8C). We further assessed the inhibitory activity of these three compounds in TNBC cells using MTT assays. The dose–response curves indicated that Echinatin exhibited the strongest anti-tumor activity, with IC_50_ values of 32.31 μM and 6.61 μM in MDA-MB-231 and MDA-MB-468 cells, respectively. Additionally, we evaluated the toxicity of Echinatin and the SPHK1 inhibitor FTY720 in normal human mammary epithelial cells (MCF-10A). Echinatin showed low toxicity, causing no significant cell death at concentrations up to 20 μM, whereas FTY720 exhibited strong toxicity at 40 μM (Figure 8D,E). Based on these results, subsequent combination therapy experiments with Niraparib were conducted using Echinatin and FTY720 within the concentration range of 0–20 μM.

As shown in the matrix dose–response plots of inhibition rates (Figure 8F,G), the synergistic effects between Niraparib and Echinatin or FTY720 were evaluated in two TNBC cell lines. The 3D synergy plots generated by SynergyFinder revealed distinct red regions indicating synergistic dose ranges. A synergy score > 10 signifies strong synergy, a score between 0 and 10 indicates additive or synergistic effects, and a score < 0 suggests antagonism. Additionally, drug synergy was assessed using CompuSyn 1.0.1, where the x-axis (Fa) represents the fraction of cells affected and the y-axis (CI) denotes the combination index (CI < 1: synergy; CI = 1: additive; CI > 1: antagonism). The results demonstrated that the highest combined doses of Niraparib and Echinatin achieved approximately 78% inhibition, with synergy scores between 0 and 10 and nearly all CI values below 1. The CI values at ED50 were also below 1 in both cell lines (Table 1). The combination reduced the IC50 of Niraparib to 18.32/9.37 μM—a more than 5-fold decrease in MDA-MB-468 cells—and that of Echinatin to 9.16/4.68 μM (Table 2). Similarly, the combination of Niraparib and FTY720 resulted in over 80% inhibition, reaching 87% in MDA-MB-468 cells, with CI values at ED50 also below 1 in both cell lines (Table 1). The IC50 of Niraparib was reduced to 8.11/9.47 μM—a more than 4-fold decrease in MDA-MB-231 and over 5-fold in MDA-MB-468 cells—while that of FTY720 decreased to 4.05/4.87 μM (Table 2). These findings indicate that Niraparib combined with Echinatin or FTY720 exhibits significant synergistic effects in TNBC cells.

Given the synergistic inhibitory effects of niraparib combined with FTY720 or Echinatin on TNBC cells in vitro, we further evaluated the anti-tumor efficacy of these combination regimens in a 4T1 tumor-bearing mouse model. Mice received daily intraperitoneal injections for 14 consecutive days at the following doses: Niraparib (25 mg/kg) plus FTY720 (5 mg/kg), or Niraparib (25 mg/kg) plus Echinatin (40 mg/kg). These dosing regimens were well-tolerated with no lethal toxicity observed. Both combination therapies markedly suppressed tumor growth, as evidenced by significant reductions in both tumor weight and volume (Figure 8H,I,K). Importantly, no significant loss of body weight was observed in any treatment group, indicating a favorable safety profile. Overall, the combination of niraparib with Echinatin demonstrated a superior safety profile compared to that of niraparib with FTY720 (Figure 8J).

As a natural product, Echinatin inhibits activity at the transcriptional level by targeting SP1, the upstream regulator of the SP1-SE-SPHK1 axis. Moreover, it exhibits a more favorable toxicity profile compared to FTY720 and demonstrates a synergistic effect when combined with Niraparib, highlighting its potential therapeutic advantage.

## 3. Discussion

The findings presented in this study unveil a previously unrecognized mechanism by which Niraparib resistance arises in TNBC through dysregulation of sphingolipid metabolism mediated by SE-driven transcriptional reprogramming. Our data demonstrate that Niraparib-induced resistance is intricately linked to the SP1-SE-SPHK1 axis, highlighting the critical interplay between epigenetic regulation and metabolic adaptation in cancer cells. These insights not only expand the understanding of Niraparib resistance mechanisms but also provide actionable therapeutic strategies to overcome this clinical challenge.

PARP proteins play multifaceted regulatory roles in lipid metabolism, involving key processes such as fatty acid synthesis, oxidation, cholesterol metabolism, and lipid storage. PARP regulates the expression of fatty acid synthases and transporters, thereby affecting fatty acid synthesis and uptake. PARP negatively regulates cholesterol synthesis by inhibiting the expression of SREBP and influences the expression of cholesterol transport proteins like ABCA1, thereby modulating cholesterol transport. Additionally, PARP proteins are involved in the regulation of adipocyte differentiation and lipid storage, impacting the occurrence of obesity and related diseases. PARP activation typically represents a destructive signal in lipid metabolism [43]. Our data show that Niraparib significantly upregulates major lipid subclasses in TNBC cells. Niraparib can significantly upregulate various lipids in cells, such as PS (phosphatidylserine), PIP2 (phosphatidylinositol 4,5-bisphosphate), PI (phosphatidylinositol), PG (phosphatidylglycerol), GM3 (ganglioside GM3), CL (cardiolipin), PC (phosphatidylcholine), LPI (lyso-phosphatidylinositol), and HexCer (hexosylceramide). The functions of these lipids in cells are complex.

We have focused on the relevant lipids in the sphingolipid metabolism pathway. For example, HexCer is a type of sphingolipid composed of sphingosine and a hexose, and it is involved in the stability of the cell membrane and signal transduction. SPH is a key intermediate in sphingolipid metabolism, and its conversion to S1P is significantly upregulated in cancer [44]. Alterations in sphingolipid metabolism can promote cancer progression, and some sphingolipids can serve as biomarkers for cancer diagnosis. For example, in breast cancer patients, the increase in C16-Cer is associated with metastatic lymph node status [45]. In addition to serving as diagnostic biomarkers, sphingolipids can also act as prognostic biomarkers for cancer. The expression levels of SPHK and S1P are related to patient survival and cancer metastasis. In esophageal cancer, high expression of SPHK1 is associated with poor 5-year overall survival (OS), and elevated SPHK1 levels are significantly correlated with metastatic lymph node status [46].

A key discovery of this work is the central role of SPHK1 upregulation in mediating Niraparib resistance. SPHK1 is highly expressed in various cancers, including breast cancer, lung cancer, head and neck cancer, and gastric cancer. A previous study conducted microarray analysis on 1269 breast cancer samples across different subtypes and found increased SPHK1 expression in cancerous tissues [47]. SPHK1, a pivotal enzyme in sphingolipid metabolism, catalyzes the conversion of sphingosine to S1P, a bioactive lipid promoting cell survival and chemoresistance [48]. Previous studies have demonstrated that SPHK1 is associated with resistance to conventional chemotherapy, and recent evidence indicates that SPHK1 enhances Olaparib resistance in ovarian cancer via the NFκB/NRF2/ferroptosis pathway [15,49]. Niraparib can promote fatty acid uptake by upregulating the expression of CD36, thereby inducing lipid peroxidation and ferroptosis [50]. These findings support our observation that co-inhibition of SPHK1 potentiates Niraparib’s ability to trigger ferroptosis. Niraparib-induced GSH depletion appears to create a “pro-ferroptosis-permissive state” in which additional stress imposed by combination partners such as FTY720—acting via the SPHK1–S1P axis on lipid metabolism—pushes cells past the point of no return into irreversible ferroptosis. Thus, although FTY720 is approved for multiple sclerosis, it displays a robust ferroptosis-inducing capacity in TNBC, underscoring its anti-tumor potential.

However, the role of SPHK1 in Niraparib resistance—particularly through SE-mediated transcriptional activation—has not been reported prior to this study. Our multi-omics analyses revealed that SPHK1 is regulated by SE, a class of large chromatin domains that drive high-level expression of oncogenes. This finding aligns with emerging evidence that SE frequently orchestrate pro-survival pathways in cancer [18], but to our knowledge, this is the first study linking SE activity to sphingolipid metabolic rewiring under PARPi treatment.

We employed a relatively novel approach by using molecular docking to predict the binding mode between the protein (SP1) and DNA (SE), and further robustly demonstrated that Niraparib can promote the binding of SP1 to the SE sequence through ChIP-qPCR. Mechanistically, we elucidated that Niraparib disrupts the PARylation of SP1, a transcription factor with established roles in stress response and oncogenesis. A critical regulatory relationship exists between SP1 and PARylation. Previous studies have shown that PARP-1 can modify SP1 through PARylation, thereby suppressing its transcriptional activity. In hepatocellular carcinoma cells, PARP-1 activation inhibits the SP1 signaling pathway to promote cell proliferation, whereas PARP-1 inhibition enhances SP1-mediated expression of checkpoint proteins (e.g., p21 and p27), leading to cell cycle arrest at the G0/G1 phase. Furthermore, PARylation inhibits SP1-driven transcription by preventing SP1 from binding to its target elements in gene promoters [37,51]. This study demonstrates that Niraparib inhibits PARylation of SP1, promotes nuclear translocation of both SP1 and the super-enhancer core protein BRD4, and facilitates the occupancy of SP1 at the super-enhancer region adjacent to the SPHK1 promoter, thereby activating its transcriptional activity and upregulating SPHK1 expression. The subsequent activation of SPHK1 enhances pro-survival signaling pathways such as PI3K/AKT and MAPK, enabling TNBC cells to escape Niraparib-induced cell death. Importantly, these observations bridge two previously overlooked aspects of Niraparib resistance: metabolic plasticity and super-enhancer-dependent transcriptional addiction. Although direct functional evidence—such as SP1 KO/KD coupled to an SE reporter assay—would provide the most compelling support, the convergence of our correlative data (ChIP-qPCR showing SP1 enrichment at the SE, siRNA-mediated disruption of super-enhancer activity, and co-localization of SP1 with BRD4) collectively points to the existence of an SP1–Super-Enhancer–SPHK1 axis.

The application of AI in drug discovery has transitioned from proof-of-concept to active clinical pipelines, encapsulated by four key themes: target discovery, molecular generation, drug repurposing, and clinical acceleration. AlphaFold can predict protein–ligand complexes at near-atomic accuracy even in the absence of experimental structures, thereby furnishing virtual screening with ready-to-use “druggable pockets.” Platforms such as PandaOmics (developed by Insilico Medicine, Cambridge, MA, USA) integrate multi-omics data—including transcriptomic and proteomic profiles—with patent and scientific literature, demonstrating practical utility by advancing a novel anti-fibrotic target, TNIK, to Phase II clinical trials within just 18 months [52]. DrugFlow is a cloud-based, all-in-one AI drug discovery platform that integrates tools such as molecular docking, QSAR, generative AI, and ADMET prediction into an intuitive interface, facilitating efficient AI-driven drug discovery [42]. We employed these platforms to conduct virtual screening for inhibitors targeting SP1.

Although Mithramycin A is currently the most extensively studied SP1 inhibitor, its clinical application is limited by dose-dependent hepatotoxicity and potential hemorrhagic risks. Administration in adults at a dose of 25 μg/kg/day for only two days has been associated with asymptomatic grade 4 elevations in ALT/AST, with peak levels reaching 2000–10,000 U/L [53]. Therefore, the development of novel SP1 inhibitors is crucial to overcome these limitations.

FTY720 has been extensively studied over the past decade, with numerous in vitro and in vivo studies confirming its activity against a wide range of solid and hematological malignancies. It can resensitize resistant cells to cetuximab and radiotherapy, prolong survival in murine models, and exhibits strong synergistic potential with existing therapies [54]. Although associated with immunosuppressive side effects, these limitations can be mitigated through structural optimization and targeted delivery strategies, positioning FTY720 as a promising candidate for drug repurposing in oncology clinical applications. In contrast, Echinatin, a natural compound derived from licorice root, has long been used as a food additive with low toxicity and minimal effects on normal cells. Its antitumor efficacy has been demonstrated across various solid tumor models, including bladder cancer, esophageal carcinoma, osteosarcoma, and liver cancer [55,56,57,58]. Consistent with our in vitro findings, the in vivo data robustly confirm that the synergistic combinations of Niraparib with FTY720 or Echinatin translate into significant anti-tumor efficacy in pre-clinical TNBC models. Among them, the Niraparib plus Echinatin regimen emerges as a promising candidate and warrants further development.

## 4. Materials and Methods

### 4.1. Compounds and Reagents

The following inhibitors were used: Olaparib (Pubchem CID:23725625, GlpBio, GC17580, Montclair, CA, USA), Niraparib (Pubchem CID:24958200, GlpBio, GC17802), Rucaparib (Pubchem CID:9931954, GlpBio, GC15955), Talazoparib tosylate (Pubchem CID:135565654, GlpBio, GC37728), FTY720 (Pubchem CID: 107969, GlpBio, GC14807), JQ1 (Pubchem CID: 46907787, GlpBio, GC13822), OTX-015 (Pubchem CID: 9936746, GlpBio, GC17973), Mithramycin A (Pubchem CID: 163659, GlpBio, GC15060), Echinatin (Pubchem CID: 6442675, GlpBio, GC38226), Helicid (Pubchem CID: 12896796, GlpBio, GN10643), Gentisic acid (Pubchem CID: 3469, GlpBio, GD20940).

The following antibodies were used: Anti-SPHK1(1:1000, proteintech, Lot No: 10670-1-AP); Anti-Erk1/2(1:1000, proteintech, Lot No: 11257-1-AP); Anti- Phospho-ERK1/2 (Thr202/Tyr204) (1:1000, proteintech, Lot No: 80031-1-RR); Anti-AKT (1:1000, proteintech, Lot No: 10176-2-AP); Anti- Phospho-AKT (Ser473) (1:1000, proteintech, Lot No: 66444-1-Ig); Anti-GAPDH (1:1000, proteintech, Lot No: 60004-1-IG); Anti-SP1 (1:1000, proteintech, Lot No: 21962-1-AP); Anti-BRD4 (1:1000, proteintech, Lot No: 67374-2-IG); Anti-PAR (1:1000, cell signaling technology, Lot No: #79109); Anti-mouse IgG, HRP-linked Antibody (1:5000, CST, Cat# 7076, Lot No: 36) and Anti-rabbit IgG, HRP-linked Antibody (1:5000, CST, Cat# 7074, Lot No: 31).

### 4.2. Cell Culture

The TNBC cell lines, including MDA-MB-231, MDA-MB-453, and MDA-MB-468, were procured from the Cell Bank of the Shanghai Institute of Biochemistry and Cell Biology (SIBCB, Shanghai, China). Additionally, the HCC1937 breast cancer cell line was acquired from Guangzhou Cellcook Biotechnology (Guangdong, Guangzhou, China). MDA-MB-231 (ER^−^/PR^−^/HER2^−^, BRCA1 wild-type), MDA-MB-468 (ER^−^/PR^−^/HER2^−^, BRCA1 wild-type), and HCC1937 (ER^−^/PR^−^/HER2^−^, BRCA1-mutant [5382insC]) were cultured in RPMI-1640 medium (Sigma-Aldrich, St. Louis, MO, USA, R8758) supplemented with 10% fetal bovine serum (FBS; Gibco, 10099141) and 1% penicillin-streptomycin (HyClone, Logan, UT, USA, SV30010). MDA-MB-453 cells (ER^−^/PR^−^/HER2^−^, BRCA1 wild-type) were maintained in DMEM/high glucose medium (HyClone™, SH30022.01) with 10% FBS and 1% penicillin-streptomycin. MCF-10A human normal mammary epithelial cells were purchased from Wuhan Sanei Biological Technology Co., Ltd. (Wuhan, China). Cells were cultured as adherent monolayers at 37 °C in a humidified atmosphere containing 5% CO_2_ and saturated humidity. Culture was maintained using the specialized MCF-10A growth medium. 4T1 murine breast cancer cells (BALB/c background) were purchased from Procell Life Science & Technology Co., Ltd. (Wuhan, China). Cells were maintained as adherent cultures at 37 °C in a humidified atmosphere of 5% CO_2_ and 95% humidity. RPMI-1640 medium was used for culture. All cell lines were authenticated by short tandem repeat (STR) profiling within six months prior to experimentation and incubated at 37 °C under 5% CO_2_.

### 4.3. Lipidomics Sequencing Analysis

Lipidomics is a research paradigm that systematically analyzes the composition and expression changes in lipids in biological entities based on high-throughput analytical techniques. Initially, experimental samples are collected. After treating triple-negative breast cancer cells (MDA-MB-231) with Niraparib (25 μM) for 48 h, samples from both the control and experimental groups are collected and processed for separation. An appropriate amount of sample is taken, and 200 μL of water and 20 μL of internal lipid standard mixture are added. The mixture is vortexed, followed by the addition of 800 μL of MTBE and vortexing again. Then, 240 μL of pre-chilled methanol is added, and the mixture is vortexed once more. The sample is then sonicated in an ice bath for 20 min, followed by incubation at room temperature for 30 min. Afterward, the sample is centrifuged at 14,000× *g* for 15 min at 10 °C. The upper organic phase is collected and dried under nitrogen. For mass spectrometry analysis, the dried sample is reconstituted in 200 μL of 90% isopropanol/acetonitrile solution, vortexed thoroughly, and then 90 μL of the solution is centrifuged again at 14,000× *g* for 15 min at 10 °C. The supernatant is then injected for analysis. The samples are separated using the UHPLC Nexera LC-30A ultra-high-performance liquid chromatography system. Mass spectrometry analysis is performed using the Q Exactive series mass spectrometer (Thermo Fisher, Waltham, MA, USA). LipidSearch software (version 4.2.28) is employed for peak identification, peak extraction, lipid identification (second-level identification), and other processing of lipid molecules and internal standard lipid molecules, to obtain the absolute quantities of lipid molecules in the samples on a large scale. LipidSearch software can achieve integrated analysis, including raw data processing, peak extraction, lipid identification, peak alignment, and quantification.

### 4.4. Transcriptomics Sequencing Analysis

Total RNA was isolated from harvested cells using the Beyoool™ Reagent (Beyotime Biotechnology, Shanghai, China, R0011), followed by enrichment of poly(A) mRNA using the NEBNext^®^ Poly(A) mRNA Magnetic Isolation Module (NEB, E7490). Purified mRNA was reverse-transcribed into cDNA using SuperScript™ IV Reverse Transcriptase (Invitrogen, Carlsbad, CA, USA, 18090010) and oligo(dT)20 primers. To construct the sequencing library, cDNA was fragmented enzymatically and ligated to Illumina TruSeq^®^ adapters (Illumina, San Diego, CA, USA, 20015965) using the NEBNext^®^ Ultra™ II DNA Library Prep Kit (New England Biolabs, Ipswich, MA, USA, E7645), with dual-index barcodes added via an 8-cycle PCR amplification. The library was quantified using Qubit™ fluorometry (Thermo Fisher, Waltham, MA, USA) and sequenced on the Illumina NovaSeq 6000 platform with paired-end sequencing (150 bp, ~40 million reads/sample). Raw sequencing data were assessed for quality using FastQC and aligned to the human reference genome (GRCh38) using STAR (v2.7.9a). Transcript quantification was performed using featureCounts (v2.0.3). Differential gene expression analysis was conducted using DESeq2 with stringent thresholds (adjusted *p*-value < 0.05, absolute log2 fold-change > 1). Functional annotation of differentially expressed genes was performed using DAVID Bioinformatics Resources (v2023q1), complemented by KEGG pathway enrichment analysis.

### 4.5. RT-qPCR Experiment

Total RNA was extracted from cell samples using the HighPure RNA Purification Kit (heruibio, HRQ0151, Fuzhou, China), with on-column DNase I treatment to eliminate genomic DNA contamination. RNA integrity was verified by agarose gel electrophoresis (28S/18S rRNA ratio > 1.8), and RNA concentration was measured using a Nanodrop 2000 spectrophotometer (Thermo Fisher, Waltham, MA, USA). Subsequently, 1 μg of RNA was reverse-transcribed into cDNA using the HiScript^®^ II Q RT SuperMix (Vazyme, Nanjing, China, R223-01). The cDNA was mixed with ChamQ Universal SYBR qPCR Master Mix (Vazyme, Q711-02) and loaded onto an RT^2^Profiler PCR Array plate (Qiagen, PAHS-343ZA, Hilden, Germany). Amplification was performed under standardized conditions on a LightCycler^®^ 96 system (Roche, 04729692001, Mannheim, Germany): one cycle of 95 °C for 30 s, followed by 40 cycles of 95 °C for 10 s, 60 °C for 30 s, and a melting curve analysis (95 °C for 15 s, 60 °C for 60 s, 95 °C for 15 s). Target validation was performed using custom primers (Sangon Biotech, Shanghai, China) with the same SYBR reagent and cycling parameters. Data were normalized to actin and analyzed using the 2^−ΔΔCt^ method. Primers used for fluorescence real-time quantitative PCR experiments are as follows Table 3.

### 4.6. Assessment of GSH Levels and MDA Content

Cells were cultured in 6-well plates under standard conditions (37 °C, 5% CO_2_) and harvested by trypsinization after treatment. The levels of intracellular reduced glutathione (GSH) and oxidized glutathione (GSSG) were determined using the GSH and GSSG Assay Kit (Beyotime Biotechnology, S0053, Shanghai, China). For detailed procedures, refer to the manufacturer’s instructions. Similarly, the required samples were collected for the experiment, and the relative levels of malondialdehyde (MDA) were measured using the Lipid Peroxidation MDA Assay Kit (Beyotime Biotechnology, S0131S, Shanghai, China). This kit employs a colorimetric reaction based on the formation of a red-colored product from the reaction of MDA with thiobarbituric acid (TBA). The MDA content in cell lysates is then quantified by spectrophotometry. This method is widely used for the detection of lipid peroxidation levels.

### 4.7. Measurement of Lipid Peroxidation

Intracellular lipid peroxidation levels were assessed using the fluorescent probe C11 BODIPY 581/591 (Thermo Fisher Scientific, Waltham, MA, USA). Briefly, cells subjected to the indicated treatments were harvested, washed with phosphate-buffered saline (PBS), and then incubated with 5 µM C11 BODIPY 581/591 in serum-free medium at 37 °C for 30 min in the dark. Following incubation, the cells were washed twice with cold PBS to remove excess probe. The fluorescence intensity of the stained cells was immediately analyzed using a flow cytometer. The probe exhibits a shift in fluorescence emission from red (approximately 590 nm) to green (approximately 510 nm) upon oxidation by lipid hydroperoxides. Data were analyzed using FlowJo software (version 10.8.1).

### 4.8. Western Blot

Total protein extracts were prepared using RIPA lysis buffer (Beyotime Biotechnology, Shanghai, China, P0013B) supplemented with protease and phosphatase inhibitors (Beyotime Biotechnology, P1049). Protein concentrations were determined using the BeyoGold™ BCA Protein Assay Kit (Beyotime Biotechnology, P0012S) according to the manufacturer’s instructions. Equal amounts of protein (20–30 μg) were separated by SDS-PAGE and transferred to methanol-activated polyvinylidene fluoride (PVDF) membranes (Beyotime Biotechnology, P0965). The membranes were blocked with 5% non-fat milk in Tris-buffered saline containing 0.1% Tween-20 (TBST) for 1 h at room temperature, followed by incubation with primary antibodies diluted in primary antibody dilution buffer (Beyotime Biotechnology, P0023A) overnight at 4 °C. After three washes with TBST (5 min each), the membranes were incubated with secondary antibodies for 1 h at room temperature. Following three additional TBST washes, protein bands were visualized using BeyoECL Plus substrate (Beyotime Biotechnology, P0018M) and quantified by densitometry using ImageJ software (version 1.53q).

### 4.9. Cell Viability Assay

Cells were seeded at a density of 5000 cells per well in a 96-well plate and allowed to adhere overnight. After stable attachment, experimental compounds were applied to the culture medium at various concentrations. Following incubation at 37 °C with 5% CO_2_ for 72 h, 20 μL of MTT reagent (5 mg/mL in phosphate-buffered saline) was added to each well. Cells were further incubated for 4 h to facilitate the mitochondrial-dependent reduction in MTT to insoluble purple formazan crystals. The culture medium was then carefully aspirated, and 150 μL of dimethyl sulfoxide (DMSO) was added to dissolve the formazan precipitate. Absorbance was measured at 570 nm using a microplate spectrophotometer. Data are presented as the mean ± standard deviation of three independent experiments.

### 4.10. siRNA Transfection

Gene-targeting siRNAs were chemically synthesized by Shanghai GenePharma Co., Ltd. (Shanghai, China). One day before transfection, cells were trypsinized, and the cell concentration was adjusted and counted. Cells were then plated in 6-well plates and incubated at 37 °C with 5% CO_2_ overnight. On the day of transfection, when the cell confluence reached approximately 60%, transfection was performed. Using the siRNA-mate plus Transfection Kit, 75 pmol of siRNA was transfected into TNBC cells for 10 h. Cells were harvested 10 h post-transfection for subsequent experimental analysis. siRNA sequences (GenePharma Co., Ltd., Shanghai, China) are listed in Table 4.

### 4.11. Multi-Dimensional Public Data Analysis

Anti-ferroptosis-related genes were systematically identified from the GeneCards database. Subsequently, the correlation between SPHK1 expression and clinical pathological features of breast cancer was analyzed using bc-GenExMiner v5.0. ChIP-seq data of H3K27ac/H3K4me1/H3K4me3 and DNase I-Seq data from various cell lines (MDA-MB-231, MDA-MB-468, HCC1937) were retrieved from the GEO database to analyze the regulation of SPHK1 by super-enhancers in TNBC and to visualize peaks using IGV software (version 2.17.4). The GEO database accession numbers for the H3K27ac-ChIP-seq data from the different cell lines are GSM2500251, GSM4874881, and GSM2258902, respectively. For MDA-MB-231, the GEO database accession numbers for the H3K27ac/H3K4me1/H3K4me3-ChIP-seq data and BRD4/BRD4-JQ1-ChIP-Seq data are GSM2500251, GSM5452343, GSM146822, GSM5954274, and GSM6775356, respectively. The DNase I-Seq data accession number is GSM2242136. The super-enhancer landscape was defined using the ROSE algorithm, and BRD4-mediated transcriptional dynamics were assessed by comparing ChIP-seq profiles with and without JQ1 treatment.

### 4.12. Chromatin Immunoprecipitation (ChIP)-qPCR

Chromatin immunoprecipitation was performed as follows: Cells were cross-linked with 1% formaldehyde (Sigma, St. Louis, MO, USA, #F8775) at 25 °C for 10 min and quenched with 125 mM glycine. Nuclei were isolated using NP-40 lysis buffer (10 mM Tris-HCl pH 8.0, 10 mM NaCl, 0.5% NP-40), and chromatin was fragmented into 200–500 bp fragments by sonication (6 cycles, 30 s ON/30 s OFF). Immunoprecipitation was carried out overnight at 4 °C with 5 μg of target-specific SP1 antibody or IgG isotype control (Cell Signaling Technology, Danvers, MA, USA, #2729), followed by incubation with Protein A/G magnetic beads (Thermo Fisher, #88802) for 2 h. Beads were washed sequentially with low-salt buffer (20 mM Tris-HCl pH 8.0, 150 mM NaCl, 0.1% SDS), high-salt buffer (500 mM NaCl), LiCl buffer (250 mM LiCl, 1% NP-40), and TE buffer. Cross-linking was reversed by incubation with 200 mM NaCl at 65 °C for 6 h, followed by protease K digestion (Thermo Fisher, #AM2548). Purified DNA was analyzed by qPCR using SYBR Green Master Mix and primers flanking the SE region (primer sequences are shown in Section 2.5). Input DNA was used as a normalization control. Enrichment was calculated using the ΔΔCt method and expressed as fold change relative to the IgG control. Statistical significance was determined by two-tailed Student’s t-test (*n* = 3 biological replicates; * *p* < 0.05, ** *p* < 0.01, *** *p* < 0.001).

### 4.13. Co-Immunoprecipitation, Co-IP

Cell samples were collected, and total protein was extracted from the cells or tissues. The cells were lysed in a non-denaturing lysis buffer to preserve the interactions between proteins. Then, specific antibodies were added to the protein lysate and incubated for a period of time to allow the antibodies to bind to the target proteins. Next, magnetic beads conjugated to the antibodies were added, and the target proteins and their interacting partners were precipitated using a magnetic force. The precipitates were then washed multiple times with a washing buffer to remove non-specifically bound proteins. Finally, the precipitated protein complexes were separated by SDS-PAGE, and the presence of the target proteins and their interacting partners was detected by Western Blot to verify the protein–protein interactions.

### 4.14. Immunofluorescence (IF) Experiment

Cells were fixed in 4% paraformaldehyde (Beyotime Biotechnology, Shanghai, China, P0099) in PBS for 15 min at 25 °C, permeabilized with 0.3% Triton X-100 (Sigma, #T9284) for 10 min, and blocked with 5% BSA at room temperature for 1 h. Primary antibodies (anti-SP1/BRD4, diluted 1:200) were incubated overnight at 4 °C in a humidified chamber, followed by three 5 min washes with PBS-T (0.1% Tween-20). Species-matched Alexa Fluor-conjugated secondary antibodies (diluted 1:500) were incubated for 1 h at 25 °C in the dark. Nuclei were counterstained with DAPI (1 μg/mL); after a final rinse with PBS, slides were mounted with ProLong™ Diamond Antifade Mountant (Thermo Fisher, Waltham, MA, USA, #P36961) and imaged using a confocal laser scanning microscope.

### 4.15. Molecular Docking with AutoDock

AutoDock is a widely used computational tool for molecular docking studies, primarily designed to predict the binding modes and affinities of small-molecule ligands with biomacromolecules such as proteins. Preparation of Files: Preparation of protein and ligand files; setting up docking parameters, running AutoGrid to generate a grid map, which provides the necessary energy calculation basis for docking. Docking Simulation: Running AutoDock; analyzing binding modes by evaluating the binding poses and binding energies of the ligand to identify the optimal binding mode. Visualization of Results: Using PyMOL to view and analyze the binding details between the ligand and the protein.

### 4.16. Applications of AlphaFold

AlphaFold encodes the protein sequence, ligand SMILES strings, and optional ion or nucleic acid information into a unified input representation. This representation is processed by the novel Pairformer module to generate pairwise interaction features. Subsequently, a diffusion module iteratively refines the three-dimensional coordinates of all heavy atoms through direct denoising in 3D space. This diffusion process progressively and simultaneously optimizes protein side-chain conformations and ligand binding poses, enabling the model to capture induced fit effects within the binding pocket. The module also provides a confidence assessment for the predicted structure.

### 4.17. Molecular Dynamics (MD) Simulation

All-atom molecular-dynamics (MD) simulations were carried out with GROMACS 2023.2. The protein was described by the AMBER99SB-ILDN force field and water by the TIP3P model. Each system was solvated in a cubic box extending 1.2 nm beyond any solute atom, neutralized with Na^+^ or Cl^−^ counter-ions and subjected to periodic boundary conditions. Long-range electrostatics were treated with the particle-mesh Ewald (PME) method (grid spacing 0.12 nm, fourth-order interpolation); van der Waals interactions were truncated at 1.0 nm and switched smoothly from 0.8 nm. Following steepest-descent energy minimization (maximum force < 1000 kJ mol^−1^ nm^−1^), the system was equilibrated in two stages: (i) 100 ps NVT ensemble at 310 K with harmonic restraints (k = 1000 kJ mol^−1^ nm^−2^) on all heavy atoms, and (ii) 100 ps NPT ensemble at 1 bar using the Parrinello–Rahman barostat (τ_p_ = 2.0 ps, compressibility 4.5 × 10^−5^ bar^−1^). Production runs were conducted for 100 ns under NPT conditions with a 2 fs time step; bonds involving hydrogens were constrained with the LINCS algorithm. Temperature was maintained at 310 K by the v-rescale thermostat (τ_t_ = 0.1 ps). Trajectories were saved every 100 ps. All analyses (RMSD, RMSF) were performed with GROMACS built-in tools and in-house Python (version 3.11.4) scripts.

### 4.18. Syngeneic 4T1 Mammary Tumor Model

The animal experiments were approved by the Experimental Animal Welfare and Ethics Committee of Fujian Medical University. Five- to six-week-old male BALB/c mice with an initial body weight of 18–20 g were housed under specific pathogen-free (SPF) conditions at 25 °C, with 8 mice per cage. To establish a breast cancer model, 1 × 10^6^ 4T1 cells (viability > 95% as determined by trypan blue exclusion assay) suspended in 100 μL of sterile PBS were subcutaneously inoculated into the right dorsal region of each mouse. When the tumor volume reached approximately 100 mm^3^, the mice were randomly divided into 7 groups (*n* = 5 per group): control (saline), vehicle (5% DMSO + 40% PEG300 + 5% Tween 80 + 50% saline), Niraparib (25 mg/kg), FTY720 (5 mg/kg), Echinatin (40 mg/kg), Niraparib + FTY720 (25 mg/kg + 5 mg/kg), and Niraparib + Echinatin (25 mg/kg + 40 mg/kg). The respective treatments were administered via intraperitoneal injections every two days. All drugs were formulated in a vehicle consisting of 5% DMSO, 40% PEG300, 5% Tween 80, and 50% saline. Tumor volume was measured using a digital vernier caliper and calculated as length × (width/2)^2^; body weight was monitored throughout the study to assess potential drug-related toxicity.

### 4.19. Statistical Analysis

Statistical analyses were performed using SPSS version 19.0. Continuous data are presented as the mean ± standard deviation (SD). Comparisons between two groups were conducted using the independent two-sample t-test (Student’s *t*-test, with significance set at *p* < 0.05). For comparisons involving multiple groups, the homogeneity of variance was initially evaluated using Levene’s test. In cases where variances were found to be homogeneous, one-way analysis of variance (ANOVA) was employed, followed by post hoc tests such as Tukey’s honestly significant difference (HSD) or least significant difference (LSD) for pairwise comparisons. When variances were not homogeneous, the nonparametric Kruskal–Wallis H test was utilized. A *p*-value of less than 0.05 was considered to indicate statistical significance. * *p* < 0.05 indicates statistical significance, ** *p* < 0.01 indicates significant statistical significance, and *** *p* < 0.001 indicates extremely significant statistical significance.

## 5. Conclusions

In summary, the four clinically important PARP inhibitors all show limited single-agent efficacy in TNBC. Niraparib—by virtue of its broad indication, favorable pharmacokinetics, metabolic/immune-modulatory potential, and wide combination window—has become an ideal scaffold for dissecting resistance mechanisms and developing rational combination strategies.

Here, we systematically delineate a novel resistance axis to the PARP inhibitor Niraparib in TNBC. Integrating lipidomic and transcriptomic analyses revealed that Niraparib disrupts sphingolipid metabolism and up-regulates the key kinase SPHK1; database mining further linked high SPHK1 to poor breast-cancer prognosis, and genetic or pharmacologic SPHK1 inhibition potentiated Niraparib-induced ferroptosis. The driver of resistance is the SP1-SE-SPHK1 regulatory module: Niraparib enhances occupancy of the transcription factor SP1 at the SPHK1-associated super-enhancer, amplifying SPHK1 transcription, upsetting sphingolipid balance, and activating pro-survival signaling that confers drug tolerance. Leveraging AI-assisted virtual screening, we identified the natural compound Echinatin as a potent SP1 inhibitor that breaks this resistance circuit and produces marked synergistic anti-tumor activity with Niraparib. Although direct targeting of downstream SPHK1 with FTY720 also restores Niraparib sensitivity, blockade of the upstream regulator SP1 offers a more strategic therapeutic intervention. These findings not only expand our understanding of Niraparib resistance beyond the DNA-damage-repair pathway but also provide a robust, clinically actionable strategy to overcome drug resistance in TNBC.

## Figures and Tables

**Table 1:** The CI of two-drug combination treatments in the TNBC cell lines.

Cell Lines	Niraparib (μM) + FTY720 (μM) ED50	Niraparib (μM) + Echinatin (μM) ED50
MDA-MB-231	0.75	0.60
MDA-MB-468	0.65	0.64

**Table 2:** The IC50 of single-agent and two-drug combination treatments in the TNBC cell lines.

Cell Lines	Niraparib (μM)	Echinatin (μM)	FTY720 (μM)	Niraparib (μM) + Echinatin (μM)	Niraparib (μM) + FTY720 (μM)
MDA-MB-231	35.90	32.31	8.54	18.32/9.16	8.11/4.05
MDA-MB-468	52.91	6.61	13.23	9.37/4.68	9.74/4.87

**Table 3:** Primer sequences used for fluorescence real-time quantitative PCR experiments.

Gene	Primer Direction	Sequence (5′ → 3′)
SPHK1-(homo)	Forward	CCTTCACGCTGATGCTCACT
	Reverse	GTTCACCACCTCGTGCATCA
SE-(homo)	Forward	CCTGTGTGACAGCCTTGGTTGAAACCC
	Reverse	GAGTGTGGACATTAGGGTCGAGAAACTC
actin-(homo)	Forward	GAGAAAATCTGGCACCACACC
	Reverse	GGATAGCACAGCCTGGATAGCAA

**Table 4:** siRNA sequences.

Gene	Direction	Sequence (5′ → 3′)
SPHK1-Homo	sense	GCGUCAUGCAUCUGUUCUATT
	antisense	UAGAACAGAUGCAUGACGCTT
SE-Homo	sense	GAUGUUGCCCAUGGAGCAATT
	antisense	UUGCUCCAUGGGCAACAUCTT
SP1-Homo	sense	GCCGUUGGCUAUAGCAAAUTT
	antisense	AUUUGCUAUAGCCAACGGCTT

## Data Availability

All data and associated protocols are included in the manuscript and available to the readers. Cell lines generated in this study are available upon request.

## Supplementary Materials

The following supporting information can be downloaded at: https://www.mdpi.com/article/10.3390/ph18111622/s1, Figure S1: The Expression of SPHK1 Is Significantly Elevated in TNBC Patient Samples and Is Negatively Correlated with Patient Prognosis; Figure S2: Despite High-Affinity Binding to SP1, Mithramycin A Exhibits Strong Off-Target Toxicity and Limited Anti-Tumor Efficacy; Table S1: Characteristics of compounds virtually screened against SP1 protein using AI technology.

## Abbreviations

Full name	abbreviation
PARP inhibitors	PARPi
homologous recombination deficiency	HRD
super-enhancers	SEs
triple-negative breast cancer	TNBC
pathological complete response rate	pCR
prolonging progression-free survival	PFS
Sphingosine Kinase 1	SPHK1
sphingosine	SPH
sphingosine-1-phosphate	S1P
transcription factors	TFs
Artificial intelligence	AI
short tandem repeat	STR
fetal bovine serum	FBS
glutathione	GSH
oxidized glutathione	GSSG
malondialdehyde	MDA
thiobarbituric acid	TBA
polyvinylidene fluoride	PVDF
Tris-buffered saline containing 0.1% Tween-20	TBST
quantitative PCR	qPCR
dimethyl sulfoxide	DMSO
Chromatin immunoprecipitation	ChIP
Co-Immunoprecipitation	Co-IP
Immunofluorescence	IF
molecular dynamics	MD
standard deviation	SD
honestly significant difference	HSD
least significant difference	LSD
Genomics of Drug Sensitivity in Cancer	GDSC
saturated fatty acids	SFAs
monounsaturated fatty acids	MUFAs
polyunsaturated fatty acids	PUFAs
Fingolimod	FTY720
multiple sclerosis	MS
sphingomyelin	SM
hexosylceramides	HexCer
ceramide	Cer
root-mean-square deviation	RMSD
root-mean-square fluctuation	RMSF
secondary structure elements	SSEs
phosphatidylserine	PS
phosphatidylinositol 4,5-bisphosphate	PIP2
phosphatidylinositol	PI
phosphatidylglycerol	PG
ganglioside GM3	GM3
cardiolipin	CL
phosphatidylcholine	PC
lyso-phosphatidylinositol	LPI
