# Supplementary material for: The SP1-SuperEnhancer-SPHK1 Axis Mediates Niraparib Resistance in TNBC
Source: Pharmaceuticals (Basel). 2025 Oct 27;18(11):1622. doi: 10.3390/ph18111622 (PMC12655459; doi:10.3390/ph18111622)
Supplement: Supplementary file 1 [file pharmaceuticals-18-01622-s001.zip › Supplement materials.docx]

**Supplement materials**

*1. The Expression of SPHK1 Is Significantly Elevated in TNBC Patient Samples and Is Negatively Correlated with Patient Prognosis.*

Using the breast cancer microarray data and RNA-seq data integrated in the bc-GenExMiner v5.0 database, we examined the correlation between SPHK1 expression levels and clinical pathological features. The results showed that SPHK1 expression was significantly higher in TNBC patients than in non-TNBC patients, regardless of whether microarray data or RNA-seq data was used. Moreover, high SPHK1 expression was associated with poor prognosis in TNBC patients (Figure S1A-B). Furthermore, we analyzed the relationship between the presence or absence of distant metastasis and SPHK1 gene expression in breast cancer patients using the TCGA database. The data revealed that breast cancer patients with distant metastasis exhibited higher expression of the SPHK1 gene (Figure S1C).

**
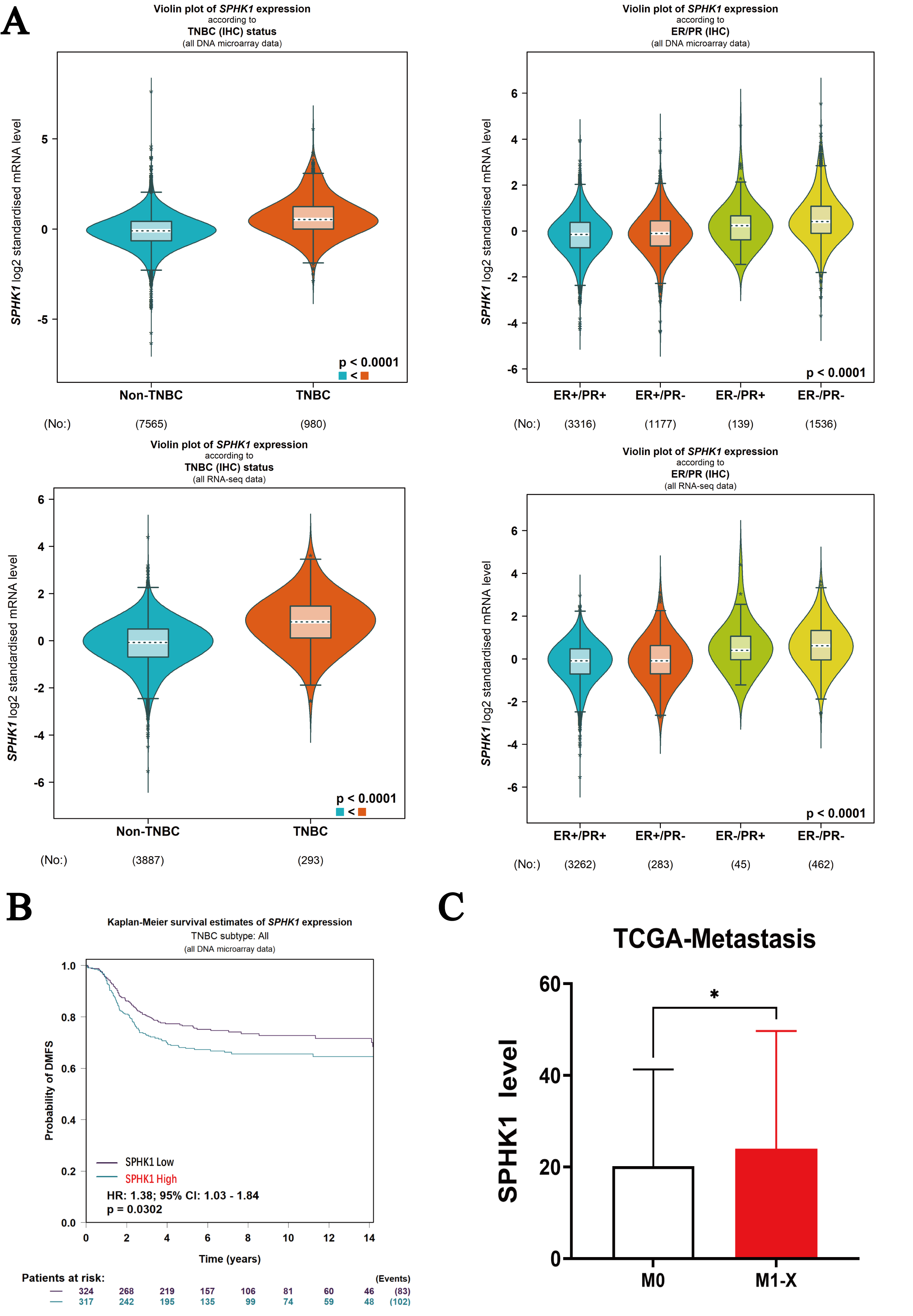
**

**Figure S1. SPHK1 gene expression is significantly upregulated in TNBC patient samples, and it is negatively correlated with patient prognosis.**

**A.** Analysis of SPHK1 expression in TNBC patient samples using clinical breast cancer databases. **B.** Correlation analysis between SPHK1 expression and prognosis in TNBC patients. **C.** TCGA-based analysis of SPHK1 expression and distant metastasis in breast cancer patients. Statistical significance between groups is indicated (* *p* < 0.05).

*2. Despite High-Affinity Binding to SP1, Mithramycin A Exhibits Strong Off-Target Toxicity and Limited Anti-Tumor Efficacy*

Molecular docking studies have shown that the free binding energy between Mithramycin A and the SP1 protein is -6.9 kcal/mol. Mithramycin A forms hydrogen bonds (indicated by blue solid lines) with LYS-12, ARG-13, and THR-15 of the protein, ionic bonds (indicated by yellow dashed lines) with LYS-12 and ARG-13, and hydrophobic interactions (indicated by gray dashed lines) with LYS-12, ARG-13, ARG-16, and GLU-19 (Figure S1A). To specifically evaluate the anti-tumor efficacy of Mithramycin A and its toxicity toward normal mammary epithelial cells, we assessed cell viability using the MTT assay after treating TNBC cell lines (MDA-MB-231 and MDA-MB-468) and normal mammary epithelial cells (MCF-10A) with a concentration gradient of Mithramycin A for 72 hours. The results demonstrated that Mithramycin A, at a concentration as low as 30 nM, induced significant cytotoxic effects in TNBC cells. More importantly, the same concentration (30 nM) also resulted in approximately 50% inhibition of viability in normal MCF-10A cells, indicating poor cellular selectivity and strong toxicity toward normal mammary epithelium. These findings suggest that Mithramycin A is unsuitable for further in vivo studies as an SP1 inhibitor. At the cellular level, we also examined the combined effect of Mithramycin A and Niraparib. The combination of 30 nM Mithramycin A with 40 μM Niraparib for 72 hours achieved only 65%–70% cytotoxicity in TNBC cells (Figure S2D).


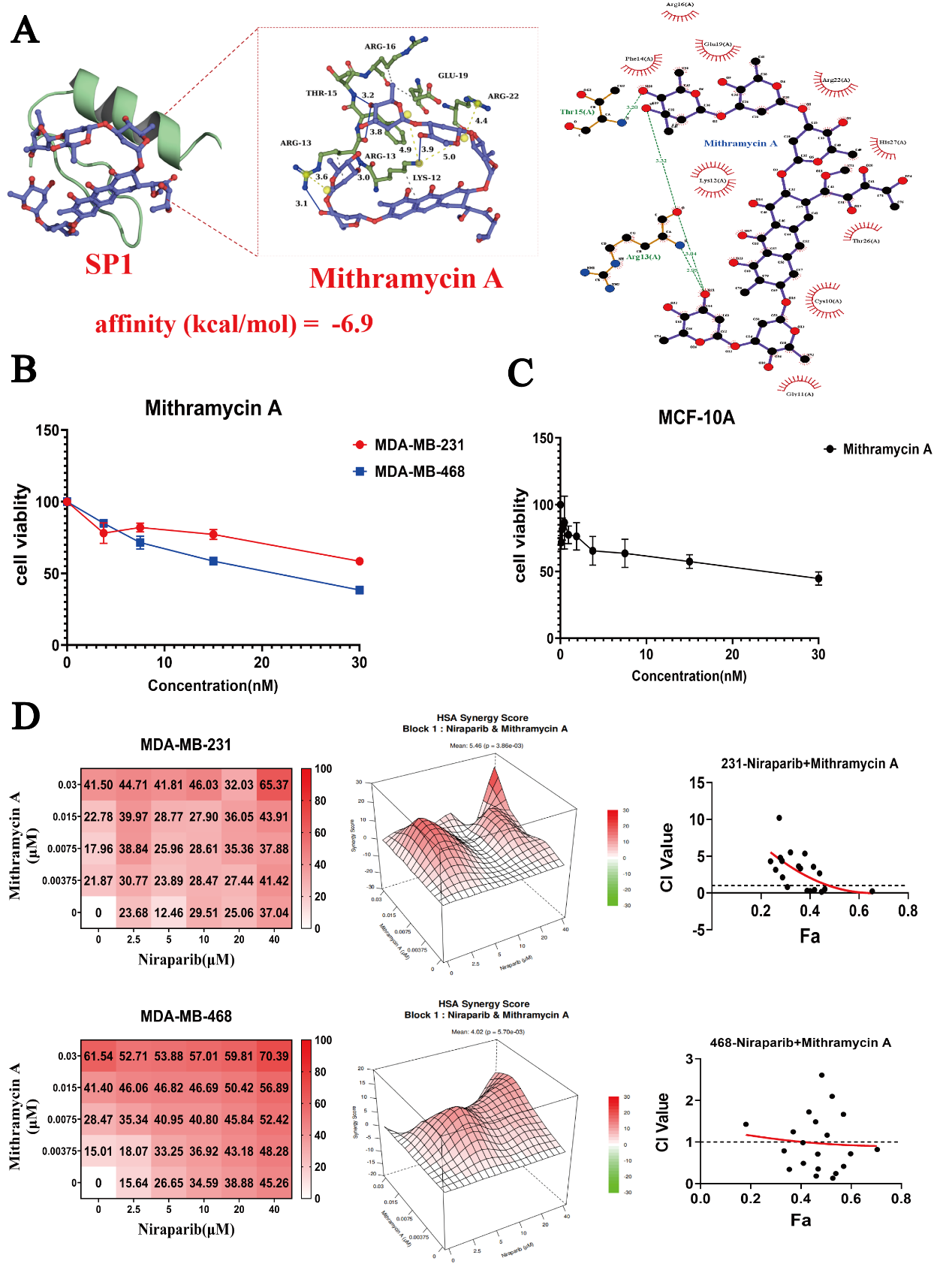


**Figure S2. Mithramycin A Binds SP1 but Lacks Cellular Selectivity and Potent Cytotoxicity.**

**A.** Molecular docking diagram of SP1 and Mithramycin A. **B-C.** Cell viability was measured by MTT assay after treatment with Mithramycin A for 72 hours in MDA-MB-231, MDA-MB-468, and MCF-10A cells. **D.** MTT assay for the effect of Mithramycin A combined with Niraparib on eliminating TNBC cells.

**Table S1. Characteristics of compounds virtually screened against SP1 protein using AI technology.**

| **RTM-rank** | **Name** | **LogP** | **RTMScore** | **autodock** | **alphafold** | **Carcinogenicity** | **DILI** | **ROA** | **hERG-10um** |  |
| --- | --- | --- | --- | --- | --- | --- | --- | --- | --- | --- |
| **1** | **Feruloyltyramine** | **2.8** | **14.23** | **-3.2** | **0.71** | **0.28** | **0.17** | **0.08** | **0.67** | **×** |
| **2** | **Oxyimperatorin** | **0.9** | **12.52** | **-4.9** | **0.8** | **0.84** | **0.88** | **0.62** | **0.38** | **×** |
| **3** | **yangonin** | **0.1** | **12.09** | **-3.9** | **0.77** | **0.74** | **0.66** | **0.32** | **0.50** | **×** |
| **4** | **Echinatin** | **2.9** | **12.06** | **-4.7** | **0.76** | **0.43** | **0.36** | **0.14** | **0.45** | **✔** |
| **5** | **Desmethoxy yangonin** | **0.1** | **11.78** | **-3.9** | **0.76** | **0.60** | **0.52** | **0.27** | **0.51** | **×** |
| **6** | **Caftaric acid** | **0** | **11.33** | **-4.7** | **0.83** | **0.06** | **0.82** | **0.08** | **0.03** | **×** |
| **7** | **Butein** | **2.8** | **11.19** | **-4.4** | **0.76** | **0.21** | **0.31** | **0.13** | **0.62** | **×** |
| **8** | **Helicid** | **-0.7** | **10.95** | **-4** | **0.72** | **0.24** | **0.34** | **0.02** | **0.21** | **✔** |
| **9** | **Benzyl benzoate** | **3.2** | **10.91** | **-4.3** | **0.46** | **0.57** | **0.28** | **0.03** | **0.84** | **×** |
| **10** | **Phthalic Acid Monooctyl Ester** | **5.5** | **10.71** | **-4.5** | **/** | **/** | **/** | **/** | **/** | **×** |
| **11** | **ISOBERGAPTEN** | **0.5** | **10.64** | **-4.4** | **0.79** | **0.82** | **0.90** | **0.62** | **0.33** | **×** |
| **12** | **3-Hydroxycoumarin** | **0.2** | **10.57** | **-4.2** | **0.77** | **0.71** | **0.65** | **0.41** | **0.37** | **×** |
| **13** | **4,4-Dimethoxychalcone** | **3.6** | **10.56** | **-4.2** | **0.78** | **0.44** | **0.79** | **0.10** | **0.47** | **×** |
| **14** | **Aegeline** | **2.4** | **10.53** | **-4** | **0.68** | **0.32** | **0.71** | **0.05** | **0.56** | **×** |
| **15** | **Leonurine** | **0.9** | **10.06** | **-3.9** | **0.86** | **0.51** | **0.20** | **0.26** | **0.82** | **×** |
| **16** | **Oxyresveratrol** | **2.7** | **9.97** | **-4.2** | **0.67** | **0.43** | **0.06** | **0.35** | **0.59** | **×** |
| **17** | **Dihydromethysticin** | **2.5** | **9.69** | **-4** | **0.82** | **0.81** | **0.86** | **0.17** | **0.57** | **×** |
| **18** | **Syringic acid** | **1** | **9.61** | **-4.2** | **0.89** | **0.42** | **0.65** | **0.32** | **0.11** | **×** |
| **19** | **Loureirin C** | **2.7** | **9.37** | **-4.3** | **0.78** | **0.45** | **0.04** | **0.28** | **0.65** | **×** |
| **20** | **2-5-dihydroxyacetophenone** | **1.4** | **9.22** | **-3.9** | **0.65** | **0.15** | **0.03** | **0.004** | **0.90** | **×** |
| **21** | **trans-Aconitic acid** | **-0.9** | **9.12** | **-4.3** | **0.89** | **0.05** | **0.99** | **0.32** | **0.001** | **×** |
| **22** | **4-Methyllimetol** | **0.4** | **9.09** | **-4.5** | **0.72** | **0.76** | **0.62** | **0.52** | **0.41** | **×** |
| **23** | **Bellidifolin** | **2.9** | **9.08** | **-3.4** | **0.78** | **0.76** | **0.62** | **0.52** | **0.41** | **×** |
| **24** | **Terephthalic acid** | **0.9** | **9.03** | **-3.9** | **0.84** | **0.32** | **0.88** | **0.28** | **0.02** | **×** |
| **25** | **Chalcone** | **3.6** | **8.99** | **-4.4** | **0.59** | **0.12** | **0.62** | **0.10** | **0.52** | **×** |
| **26** | **dencichine** | **-4** | **8.97** | **-4.1** | **0.89** | **0.10** | **0.66** | **0.22** | **0.01** | **×** |
| **27** | **5,7-Dihydroxychromone** | **0** | **8.89** | **-4.3** | **0.79** | **0.75** | **0.70** | **0.64** | **0.46** | **×** |
| **28** | **Gentisic acid** | **1.2** | **8.84** | **-4.1** | **0.84** | **0.30** | **0.35** | **0.33** | **0.15** | **✔** |
| **29** | **Methysticin** | **2.3** | **8.81** | **-4.3** | **0.8** | **0.91** | **0.88** | **0.33** | **0.39** | **×** |
| **30** | **Coumarin** | **0.2** | **8.77** | **-4** | **0.86** | **0.80** | **0.75** | **0.48** | **0.41** | **×** |
